# Working during the COVID-19 pandemic: Demands, resources, and mental wellbeing

**DOI:** 10.3389/fpsyg.2022.1037866

**Published:** 2023-01-13

**Authors:** Tabea Eleonore Scheel, Lydia Bendixen, Jakub Procházka, Daniela Acquadro Maran

**Affiliations:** ^1^Department of Work and Organizational Psychology, Europa-Universität Flensburg, Flensburg, Germany; ^2^Department of Corporate Economy, Masaryk University, Brno, Czechia; ^3^Department of Psychology, Università degli Studi di Torino, Turin, Italy

**Keywords:** COVID-19 pandemic, JD-R model, work intensification, irritation, home office

## Abstract

The aim of this study was to investigate the relationship between working conditions at the start of the COVID-19 pandemic (spring 2020) and employees’ mental wellbeing. According to the Job Demands-Resources (JD-R) model, work intensification, increased difficulty in accomplishing work tasks, heightened risk of infection by COVID-19, and increasingly working from home may detrimentally relate to irritation. However, personal and job resources (e.g., occupational self-efficacy, social support) may buffer. Data from 680 employees from four European countries were analyzed by means of path analyses and polynomial regression. Work intensification was significantly positively associated with cognitive and affective irritation; other job demands were not. However, working from home prior to as well as during the pandemic was related to higher cognitive irritation. None of the moderators was of meaningful significance. Reducing work intensification as well as enduring home office seems to be crucial for interventions.

## 1. Introduction

Being the largest public health and economic crisis in a century (Organisation for Economic Co-operation and Development [OECD], 2020), the severe acute respiratory syndrome coronavirus 2 (SARS-CoV-2) has caused a million cases of coronavirus disease in 2020 (COVID-19; WHO, 2021). By the first half of April 2020, mandatory and recommended mitigation and containment policies were non-pharmaceutical interventions, such as hygiene measures, mobility restrictions, school closures, and the interruption of non-essential business activities, introduced to protect employees and reduce further spreading of the pandemic (Foucault and Galasso, 2020). These confinement and lockdown measures brought changes to the way of working (e.g., interruptions of work activity and reduced working hours; Eurofound, 2020; Organisation for Economic Co-operation and Development [OECD], 2020). On the contrary, for many employees, these changes included an intensification of work or higher difficulties in accomplishing formerly routine work, for instance, due to novel demands and tasks. Also, between 30 and 60% of workers in OECD countries worked from home in mid-April 2020 (Foucault and Galasso, 2020); 37% of employees in Europe started teleworking due to COVID-19 (Eurofound, 2020) rather abruptly. However, two-thirds of working tasks cannot (or only with difficulty) be performed from home (e.g., healthcare work), therefore, including a heightened risk of contagion (Organisation for Economic Co-operation and Development [OECD], 2020).

Among the consequences of working in home office are loss of productivity, collisions with caregiving responsibilities, changes in work hours (e.g., Collins et al., 2020), impediment of recovery, work intensification, as well as extensification (e.g., Kelliher and Anderson, 2010; Biron and van Veldhoven, 2016), and technostress (Spagnoli et al., 2020; Camacho and Barrios, 2022). Telework is associated with stress (e.g., Lapierre et al., 2016). Overall, the work-related demands of the COVID-19 pandemic may be perceived by employees as a threat (e.g., Madero Gómez et al., 2020; Mo et al., 2020).

Accordingly, during the lockdown in the spring of 2020, mental wellbeing decreased, and loneliness and anxiety increased in Europe (Eurofound, 2020). Physical distancing reduced important social connections and the ability to cope with pandemic-related restrictions (van Bavel et al., 2020). While prior research focused on work stress and psychological experiences of healthcare workers (Mo et al., 2020; Sun et al., 2020) or the impact of COVID-health anxiety on (work) goal progress (Trougakos et al., 2020), the relation between perceived working conditions due to COVID-19 and mental wellbeing remains to be addressed by research.

We aim to answer whether job demands during the start of the COVID-19 pandemic relate to employees’ reduced mental wellbeing (irritation) and whether personal and social resources buffer these relationships.

Based on the Job Demands-Resources (JD-R) model and the Conservation of Resources Theory (COR) (Hobfoll, 1989; Demerouti et al., 2001), we propose that employees in various occupations face job demands, such as intensified work, increased work difficulty, increased working from home, and the risk of being infected by COVID-19. These demands threaten employees’ mental wellbeing if not counteracted by resources moderating the stressor–strain relation. Occupational self-efficacy (Ventura et al., 2015), emotional change readiness (Bouckenooghe et al., 2009), and job crafting (e.g., Tims et al., 2013) are relevant personal resources. Social support from colleagues may be a highly relevant social resource (Crawford et al., 2010; Kniffin et al., 2021).

Our study makes two contributions. First, job demands and resources as relevant for the start of the COVID-19 pandemic are examined within the framework of the JD-R model, thus conceptually and practically helping to understand the work-related demands. Second, against the background of the mandatory home office, we provide an in-depth analysis of the relation of working from home prior to and during the pandemic and its association with irritation by polynomial regression. The validity of the contributions is underpinned by preregistered hypotheses and data from Germany, Czechia, Slovakia, and Italy.

The health-impairment path of the JD-R model (Demerouti et al., 2001) explains why working conditions due to the COVID-19 pandemic should relate to irritation. Job demands are physical, psychological, social, or organizational characteristics of the workplace (Demerouti et al., 2001). Empirical evidence of the JD-R model is broad (e.g., Halbesleben, 2006; Crawford et al., 2010; Lesener et al., 2019). The health-impairment process (Bakker and Demerouti, 2017) states that an energy-depletion process arises when job demands are high (and the resources are limited), requiring sustained physical and/or psychological energy or abilities (cognitive and affective), leading to a lack of energy (Demerouti et al., 2001). Consequently, the associated psychological stress exhausts. Irritation, as a proximal mental health indicator of work stress, is a precursor of more serious impairments such as psychosomatic complaints and depression (e.g., Mohr et al., 2006). Irritation is a state of mental impairment resulting from a perceived goal discrepancy; its subcategory cognitive irritation, or rumination, is “a state of reinforced efforts toward goal-achievement” (Mohr et al., 2006, p. 199). The subcategory affective irritation (i.e., irritability) is a more severe state of mental strain, in which the person loses the incentive to achieve a certain goal (Mohr et al., 2006). Depleting job demands may relate to irritation as a proximal consequence (Nolen-Hoeksema et al., 2008).

Under pandemic conditions, sudden and novel changes in job demands may be rather experienced as hindrance stressors, which involve excessive or undesirable constraints, require fast adaptations, and coping strategies, and are detrimental to wellbeing (Podsakoff et al., 2007; Schaufeli et al., 2009; Crawford et al., 2010).

*Work intensification* occurs within the accelerated change of work and organizational environment (Kubicek et al., 2015). Additional efforts (increasing speed, multitasking, and fewer breaks) are needed to complete the work (Korunka et al., 2015). Due to the COVID-19 lockdown, the changes in work procedures and environment (e.g., required hygiene measures, online communication; Nakrošienë et al., 2019), *work difficulty* may have increased. The *risk of being infected* with SARS-CoV-2 during work is a stressor for employees. For instance, general health anxiety about having or contracting COVID-19 increased somatic complaints over time (Trougakos et al., 2020). If *working from home* is mandatory, potential positive effects disappear (Lapierre et al., 2016). Intensively working from home (more than 2.5 days a week) has detrimental effects on the relationships with coworkers (Gajendran and Harrison, 2007), like reduced social support due to telecommunication (Sardeshmukh et al., 2012). Reduced personal contacts, social distancing (Brooks et al., 2020), as well as disruptive task setbacks related to telework during the COVID-19 pandemic (Chong et al., 2020), may harm mental wellbeing. Thus, we hypothesize:

*Hypotheses H1*: (a) Work intensification, (b) increased work difficulty, (c) the risk of being infected by COVID-19 during work, and (d) increased working from home relate positively to cognitive and affective irritation.

However, resources may buffer the health-impairment process of hindering job demands and strain in a gain spiral (e.g., Hobfoll, 1989; Bakker and Demerouti, 2017).

*Occupational self-efficacy* is defined “(…) as the confidence an individual has in her or his ability to cope with difficult tasks or problems” (Rigotti et al., 2008; p. 239) in the work context. Occupational self-efficacy helps to cope with energy-depleting work situations and to adapt successfully to challenging circumstances. *Emotional change readiness* is said to “capture the feelings about a specific change project” (Bouckenooghe et al., 2009; p. 576) and should help employees to adapt to changes in the work situation, like rising or more difficult work tasks, infection risks, or online communication due to home office.

*Social support by colleagues*, such as work relationships in general, is a strong predictor of attitudes to organizational change (Vakola and Nikolaou, 2005); meta-analyses confirm social support as a resource attenuating the stressor–strain relationship (e.g., Crawford et al., 2010), especially when social support is work-related (Halbesleben, 2006). *Job crafting* is an employee’s self-initiated change of her/his/their job demands and resources consistent with the JD-R model (Tims et al., 2013). Increasing job resources and reducing hindering job demands were negatively related to emotional exhaustion (Lichtenthaler and Fischbach, 2016).

*Hypotheses H2–H5*: Occupational self-efficacy (H2), emotional change readiness (H3), social support by colleagues (H4), and job crafting (H5) moderate the positive relationship between (a) work intensification, (b) increased work difficulty, (c) the risk of being infected by COVID-19 during work, and (d) increased working from home and cognitive as well as affective irritation, such that the higher the [respective resource] the weaker the relationship.

## 2. Methods

This cross-sectional quantitative study is one of two preregistered studies sharing the same data collection about working conditions early in times of the COVID-19 pandemic. While the study at issue focused on wellbeing,^1^ the second study assessed (changes in) performance and commitment (see text footnote 1). The only two variables preregistered for both studies were increased work difficulty and risk of being infected by COVID-19 (i.e., working conditions, refer to the following text). Data descriptives (also by country) are available in an open-source data article (refer to Procházka et al., 2020), which also included information about scales, translations, and reliabilities, while confirmatory factor analyses (CFA) were conducted specifically for the study at issue.

The survey was administered at a time when government restrictions due to the “first wave” of the COVID-19 pandemic were in place in many European countries (May/June 2020). Using Qualtrics (except Questback for Germany), study participants were invited *via* social networks, newspaper articles, emails, and university newsletters.

A sample of *N* = 680 employees was analyzed (*n* = 138 German, *n* = 230 Czech, *n* = 161 Slovakian, *n* = 151 Italian). Participants were 444 women (65%), 227 men, 2 “other,” and 7 did not indicate. The mean age was *M* = 39.9 (*SD* = 12.3), ranging from 19 to 71. University was the most frequent degree (*n* = 459; 68%), and services for customers (*n* = 207; 30.4%), public sector (*n* = 163; 24%), and education (*n* = 117; 17%) were the most frequent sectors. For 78% of persons, the home office increased in the pandemic (21% from *never* to *fully*). For further details about the sample and dataset, refer to Procházka et al. (2020).

### 2.1. Operationalization

Established and validated scales were chosen where possible (work intensification, Intensified Job Demands Scale, Kubicek et al., 2015; occupational self-efficacy, Rigotti et al., 2008; readiness for change, affective subscale, Organizational Change Questionnaire, Bouckenooghe et al., 2009; social support from colleagues, Herrmann et al., 2012; job crafting–increasing job resources and hindering job demands, Tims et al., 2012; irritation–cognitive, affective, Mohr et al., 2006). Reliabilities ranged from Cronbach’s α = 0.78 to 0.88. Single-item measures (11-point response scales; 0–10) were chosen for increased work difficulty, risk of infection during the workday, and extent of work from home before/during the pandemic (difference score). *Increased work difficulty* was measured by one question: “How much has the pandemic increased the difficulty of your work (e.g., because of the need to wear protective equipment, because of increased hygiene measures, because of the need to communicate online)?” *The risk of infection during the workday* was assessed by one question: “How great is the risk of becoming infected with COVID-19 in the course of your work?” *More work from home (home-office)* was assessed as a difference score (as well as polynomial regressions) of two questions: “How often did you work from home before the pandemic and lockdown?” and “How often do you work from home now in the time of the pandemic and lockdown?”

### 2.2. Statistical analysis

In line with the preregistration, we tested the hypotheses using path analysis with a robust estimator (MLR) in MPLUS 8.2. In addition to the flawed difference scores, the relationship between working from home before vs. during the pandemic and irritation was tested with polynomial regression (Edwards, 1994) and response surface analyses (Shanock et al., 2010).

## 3. Results

Confirmatory factor analyses demonstrated metric invariance across countries and discriminant validity of variables that were measured by scales. The CFA on the complete sample of the study at issue supported the factor structure (Supplementary Table 1). The level of common method variance was low (single factor accounting for only 13.85% of the variance).

### 3.1. Path analysis

The model explained 29.6% of the variance in cognitive and 19.3% in affective irritation. We found support for H1a as the relationship between work intensification and cognitive vs. affective irritation was significantly positive (*B* = 0.40, *SE* = 0.03 vs. *B* = 0.30, *SE* = 0.04; *p* < 0.001; effects were also significant for each country sample). H1b was only partially supported for the (very weak) association between the increased difficulty of work and cognitive irritation. H1c and H1d were not supported, as neither the risk of being infected with COVID-19 nor the change in the home office was significantly positively associated with irritation. The rather surprisingly negative relation between infection risk and cognitive irritation was very weak and practically insignificant (Table 1).

**TABLE 1:** Path analysis with two dependent variables.

	Cognitive irritation				Affective irritation			
	***B***	***S.E.***	***p***	**β**	***B***	***S.E.***	***p***	**β**
Work intensification	0.40	0.03	<0.001	0.42	0.30	0.04	<0.001	0.31
Work difficulty	0.03	0.01	0.022	0.09	0.01	0.01	0.681	0.02
Risk of infection	-0.04	0.01	0.003	-0.11	-0.02	0.01	0.216	-0.05
ΔHome office	-0.01	0.01	0.536	-0.02	0.00	0.01	0.816	-0.01
Occupational self-efficacy	-0.19	0.05	<0.001	-0.13	-0.22	0.06	<0.001	-0.15
Readiness for change	-0.08	0.05	0.078	-0.07	-0.13	0.05	0.015	-0.10
Social support colleagues	-0.21	0.04	<0.001	-0.17	-0.18	0.05	<0.001	-0.15
Job crafting/resources	0.08	0.04	0.020	0.08	-0.03	0.04	0.444	-0.03
Job crafting/demands	-0.08	0.05	0.069	-0.06	0.10	0.05	0.036	0.08

*N* = 680. Single country sample tests of work intensification and irritation: Germany (cognitive: *B* = 0.48, *S.E.* = 0.06, *p* < 0.001; affective: *B* = 0.43, *S.E.* = 0.07, *p* < 0.001), Czechia (cognitive: *B* = 0.35, *S.E.* = 0.06, *p* < 0.001; affective: *B* = 0.26, *S.E.* = 0.06, *p* < 0.001), Slovakia (cognitive: *B* = 0.33, *S.E.* = 0.06, *p* < 0.001; affective: *B* = 0.25, *S.E.* = 0.07, *p* = 0.001), and Italy (cognitive: *B* = 0.47, *S.E.* = 0.06, *p* < 0.001; affective: *B* = 0.28, *S.E.* = 0.07, *p* < 0.001).

We conducted a series of moderation analyses each with the 10 centered predictors (i.e., 4 independent variables, 5 moderators, 1 interaction). As 40 different moderation effects in 20 different analyses were tested (in a large sample), the level of significance was set to α = 0.0025 (α = 0.05/20) to prevent finding marginal false positive effects. Overall, we did not find support for any of the moderation hypotheses (H2a–d to H5a–d). *Occupational self-efficacy* moderated the association of work intensification (a) and increased work difficulty (b) with affective irritation (H2a; β = −0.09, *p* = 0.025, Δ*R*^2^ = 0.007; H2b; β = −0.08, *p* = 0.021, Δ*R*^2^ = 0.006). *Emotional change readiness* moderated the relationship between the risk of being infected by COVID-19 and affective irritation (H3d; β = −0.07, *p* = 0.039, Δ*R*^2^ = 0.005). *Social support by colleagues* moderated the relationship between increased work from home and irritation (H4d; affective β = −0.09, *p* = 0.008, Δ*R*^2^ = 0.009; cognitive β = −0.07, *p* = 0.036, Δ*R*^2^ = 0.005). However, all these reported effects were very small, insignificant on α = 0.0025, and unstable across countries; the other moderation effects were even smaller (Supplementary Figure 1 and Supplementary Tables 2, 3).

### 3.2. Polynomial regression

**FIGURE 1:**
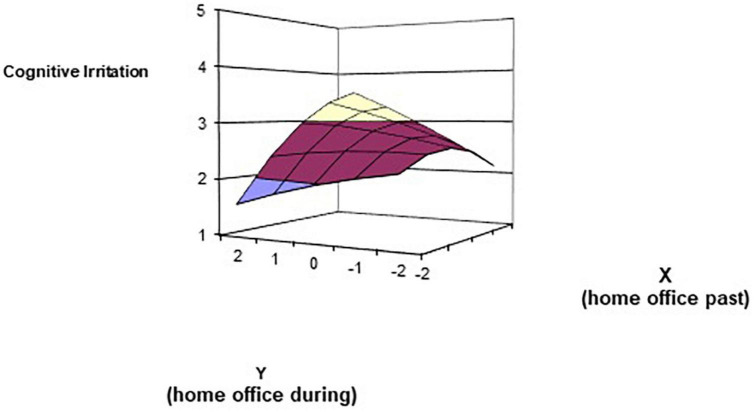
Response surface analysis of working from home prior to (X) and during (Y) the pandemic spring of 2020 in relation to cognitive irritation. Germany, Czechia, and Italy were included as dummy variables (1 = *yes*, 0 = *no*); Slovakia served as the reference country. Coefficients: a_1_ = 0.34 (*p* = 0.001); a_2_ = 0.02 (*ns*); a_3_ = 0.14 (*ns*); a_4_ = –0.24 (*p* = 0.005).

As regulations and lockdown conditions varied considerably, we controlled for the country participants indicated to work in when conducting the polynomial regressions of increase in the home office (before vs. during the pandemic). The response surface coefficients (Shanock et al., 2010) were not significant for affective irritation (a_1_ = 0.11, *p* = 0.315; a_2_ = −0.06, *p* = 0.495; a_3_ = 0.12, *p* = 0.281; a_4_ = −0.05; *p* = 0.542). For cognitive irritation (Figure 1), the coefficients for the line of perfect agreement a_1_ = 0.34 (*p* = 0.001) and the (concave) curvature along the line of incongruence a_4_ = −0.24 (*p* = 0.005) indicated that cognitive irritation (a_1_) increases linearly with a mutual increase in working from home before and during the pandemic, and (a_4_) decreases with the higher discrepancy between home office prior to vs. during the pandemic.

## 4. Discussion

Work intensification as attributed to the pandemic was significantly related to higher cognitive and affective irritation, a finding being in line with prior work on the association of work intensification with reduced mental wellbeing (e.g., Chesley, 2014). Given that work intensification is connected to burnout (Kubicek et al., 2015; Afshari et al., 2022; Venz and Boettcher, 2022) and future emotional exhaustion (Korunka et al., 2015), it also seems to be an important finding under the pandemic conditions. The other non-significant findings (work difficulty, the risk of infection, increased work from home) may indicate that work-related job demands were not associated with irritation, but they could also be attributed to a rather moderate level of irritation. Surprisingly, rather than the change into a home office (Δhome office), the overall amount of home office seems problematic (refer to the Section “3.2 Polynomial regression”). However, difference scores suffer from low reliability. In other words, “always” working from home is related to higher cognitive irritation, a finding that sheds new light on the discussion of conceding legal rights for working in home office (e.g., in Germany) to employees. Given that the increase in home office affected 80% of our sample, the non-significant finding for affective irritation was surprising. Overall, cognitive irritation was rated slightly higher than affective irritation. Maybe in the first lockdown in the spring of 2020, cognitive irritation was the more relevant mental stress variable and affective irritation only increased with the subsequent lockdowns.

None of our moderating resources buffered the relationship between increasing/novel job demands and irritation during the pandemic work situation in the spring of 2020. One reason might be the only moderate average irritation, which did not leave much room for being mitigated by resources. In line with former findings (e.g., Xanthopoulou et al., 2007; Tims et al., 2013), the resources had rather direct significant relationships with cognitive and affective irritation. Research during COVID-19 showed that perceived organizational support negatively relates to burnout (Afshari et al., 2022). Similar negative associations between social support and anxiety were also shown by a Spanish study during the first wave of COVID-19 (González-Sanguino et al., 2020).

### 4.1. Strengths and limitations

The data collection in four different European countries at a unique point in time, namely, the first wave of the COVID-19 pandemic and the related lockdown in spring 2020, is among the strengths of this study. Work intensification in the pandemic was related to irritation across four countries; the findings about the general risk of the home office for higher irritation are novel. Results are stable across samples and four countries. Also, our sample size was large, the study was pre-registered, and the questionnaires’ quality was demonstrated by the very good fit of the measurement model. Reporting all (including the non-significant) results counteracts data fishing.

However, the cross-sectional design with self-reported data collected online from a single source within a convenience sample should be considered when interpreting the study results. Despite being economical, using one-item measures does not allow the establishment of a latent variable or test reliability. However, a well-formulated item can measure a non-complex construct.

### 4.2. Implications

An intriguing question for future research is the effect of time, that is, the linear or cumulative consequences of the COVID-19-related policies and different lockdown waves on employees’ mental health. Focusing on specific occupations with, for instance, a high risk of infection by COVID-19 and less opportunity for home office would be worthwhile. Organizations and supervisors should be aware of rising work intensification in pandemics and take countermeasures (e.g., redesigning work, providing resources, and hiring additional staff). Independently from a pandemic, intensively working from home should be restricted by organizations. While offering a home office on a voluntary basis may have benefits (e.g., flexibility) for employees, the costs for mental health may be similar to those found for social relationships with coworkers who suffered when mainly working from home (Gajendran and Harrison, 2007). Furthermore, additional problems accompanying working from home, such as technostress (e.g., Camacho and Barrios, 2022), should be considered in future studies.

### 4.3. Conclusion

Pandemic-related work intensification is related to irritation, and the enduring home office seems to be a risk. However, in the first COVID-19 lockdown in Europe, working conditions and mental health state were not generally on a worrisome level. The long-term changes in working conditions and the consequences on employees’ mental health are promising avenues for future research.

## Data availability statement

The raw data supporting the conclusions of this article will be made available by the authors, without undue reservation.

## Author contributions

TS, JP, and DA contributed to conception, design of the study, and organized the database. TS and JP performed the statistical analysis. LB and TS wrote the first draft of the manuscript. TS, JP, and LB wrote sections of the manuscript. All authors contributed to manuscript revision, read and approved the submitted version.

## Footnotes

1
osf.io/9q6vt
