# Supplementary material for: Working during the COVID-19 pandemic: Demands, resources, and mental wellbeing
Source: Front Psychol. 2023 Jan 13;13:1037866. doi: 10.3389/fpsyg.2022.1037866 (PMC9880045; doi:10.3389/fpsyg.2022.1037866)
Supplement: Supplementary file 1 [file Data_Sheet_1.pdf]

Supplementary Material

Supplementary Table 1.

Measurement Invariance across Countries and Factors

	N	χ^2	df	*p*	CFI	TLI	RMSEA
Germany	138	330.98	247	<.001	.95	.94	.05 [.03, .06]
Czech Rep.	230	362.42	247	<.001	.95	.93	.05 [.04, .06]
Slovakia	161	317.76	247	.002	.96	.95	.04 [.03, .06]
Italy	151	305.65	247	.007	.96	.95	.04 [.02, .05]
Multi-group							
M1:Configural	680	1317.34	988	<.001	.95	.94	.04 [.04, .05]
M2:Metric	680	1410.19	1063	<.001	.95	.94	.04 [.04, .05]
M3:Scalar	680	1727.72	1138	<.001	.91	.91	.06 [.05, .06]
One-group[a]	680	426.99	247	<.001	.97	.97	.03 [.03, .04]

Note. Maximum likelihood estimation with robust standard errors (MLR) in MPLUS 8.2 (Muthén and Muthén, 1998-2017). CFI = Comparative Fit Index; TLI = Tucker-Lewis Index; RMSEA = Root Mean Square Error of Approximation. [a]This eight-factor model was preferred over a seven-factor model with *one* factor merging cognitive and affective irritation, $\chi^2(254)$= 978.82, $p < .001$, CFI = .88, TLI = .86, RMSEA = .07, ΔCFI = .09, over a seven-factor model with one factor of job crafting, $\chi^2(254)$= 726.05, $p < .001$, CFI = .92, TLI = .91 RMSEA = .05, ΔCFI = .05, and also over the one-factor model, $\chi^2(275)$= 4734.65, $p < .001$, CFI = .28, TLI = .22 RMSEA = .15, ΔCFI = .64.

Supplementary Table 2

Regression with demands, resources, and irritation

LEGEND

IRITC – Cognitive irritation

IRITA – Affective irritation

WI – Work intensification

WD – Change in work difficulty

COV - Risk of being infected by COVID-19 during workday

HO – Change in home office

SE – Occupational self-efficacy

RC – Emotional readiness for change

SS – Social support by colleagues

JCR - Job crafting – increasing resources

JCD - Job crafting – hindering demands

X - Symbol for interaction

SYNTAX

ANALYSIS:

ESTIMATOR IS MLR;

MODEL:

IRITC on WI WD COV HO SE RC SS JCR JCD;

IRITA on WI WD COV HO SE RC SS JCR JCD;

MODEL RESULTS

	Estimate	S.E.	Est./S.E.	Two-Tailed P-Value
IRITC ON				
WI	0.399	0.033	12.113	0.000
WD	0.028	0.012	2.286	0.022
COV	-0.037	0.013	-2.945	0.003
HO	-0.006	0.009	-0.620	0.536

SE	-0.194	0.052	-3.703	0.000
RC	-0.083	0.047	-1.765	0.078
SS	-0.205	0.039	-5.202	0.000
JCR	0.083	0.036	2.321	0.020
JCD	-0.084	0.046	-1.818	0.069

IRITA ON

WI	0.298	0.038	7.859	0.000
WD	0.005	0.013	0.411	0.681
COV	-0.016	0.013	-1.238	0.216
HO	-0.002	0.010	-0.233	0.816
SE	-0.220	0.059	-3.697	0.000
RC	-0.129	0.053	-2.429	0.015
SS	-0.176	0.047	-3.760	0.000
JCR	-0.030	0.040	-0.766	0.444
JCD	0.103	0.049	2.096	0.036

IRITA WITH

IRITC	0.230	0.032	7.090	0.000

Intercepts

IRITC	2.780	0.033	83.341	0.000
IRITA	2.438	0.036	68.234	0.000

Residual Variances

IRITC	0.757	0.039	19.614	0.000
IRITA	0.867	0.041	21.136	0.000

STANDARDIZED MODEL RESULTS

STDYX Standardization

	Estimate	S.E.	Est./S.E.	Two-Tailed P-Value
IRITC ON				
WI	0.418	0.033	12.685	0.000
WD	0.085	0.037	2.287	0.022
COV	-0.108	0.037	-2.937	0.003
HO	-0.022	0.035	-0.620	0.535
SE	-0.134	0.036	-3.686	0.000
RC	-0.066	0.038	-1.764	0.078
SS	-0.171	0.033	-5.164	0.000
JCR	0.081	0.035	2.316	0.021
JCD	-0.063	0.035	-1.816	0.069
IRITA ON				
WI	0.312	0.039	8.026	0.000
WD	0.016	0.039	0.411	0.681
COV	-0.048	0.039	-1.238	0.216
HO	-0.009	0.038	-0.233	0.816
SE	-0.151	0.041	-3.689	0.000
RC	-0.102	0.042	-2.426	0.015
SS	-0.147	0.039	-3.764	0.000
JCR	-0.030	0.039	-0.765	0.444
JCD	0.077	0.037	2.098	0.036
IRITA WITH				
IRITC	0.284	0.037	7.712	0.000
Intercepts				
IRITC	2.681	0.063	42.317	0.000
IRITA	2.348	0.050	46.718	0.000
Residual Variances				
IRITC	0.704	0.029	23.857	0.000
IRITA	0.804	0.028	28.803	0.000

R-SQUARE

Observed Variable	Estimate	S.E.	Est./S.E.	Two-Tailed P-Value
IRITC	0.296	0.029	10.046	0.000
IRITA	0.196	0.028	7.028	0.000

Supplementary Figure 2. Path diagram with standardized coefficients of demands, resources, and irritation

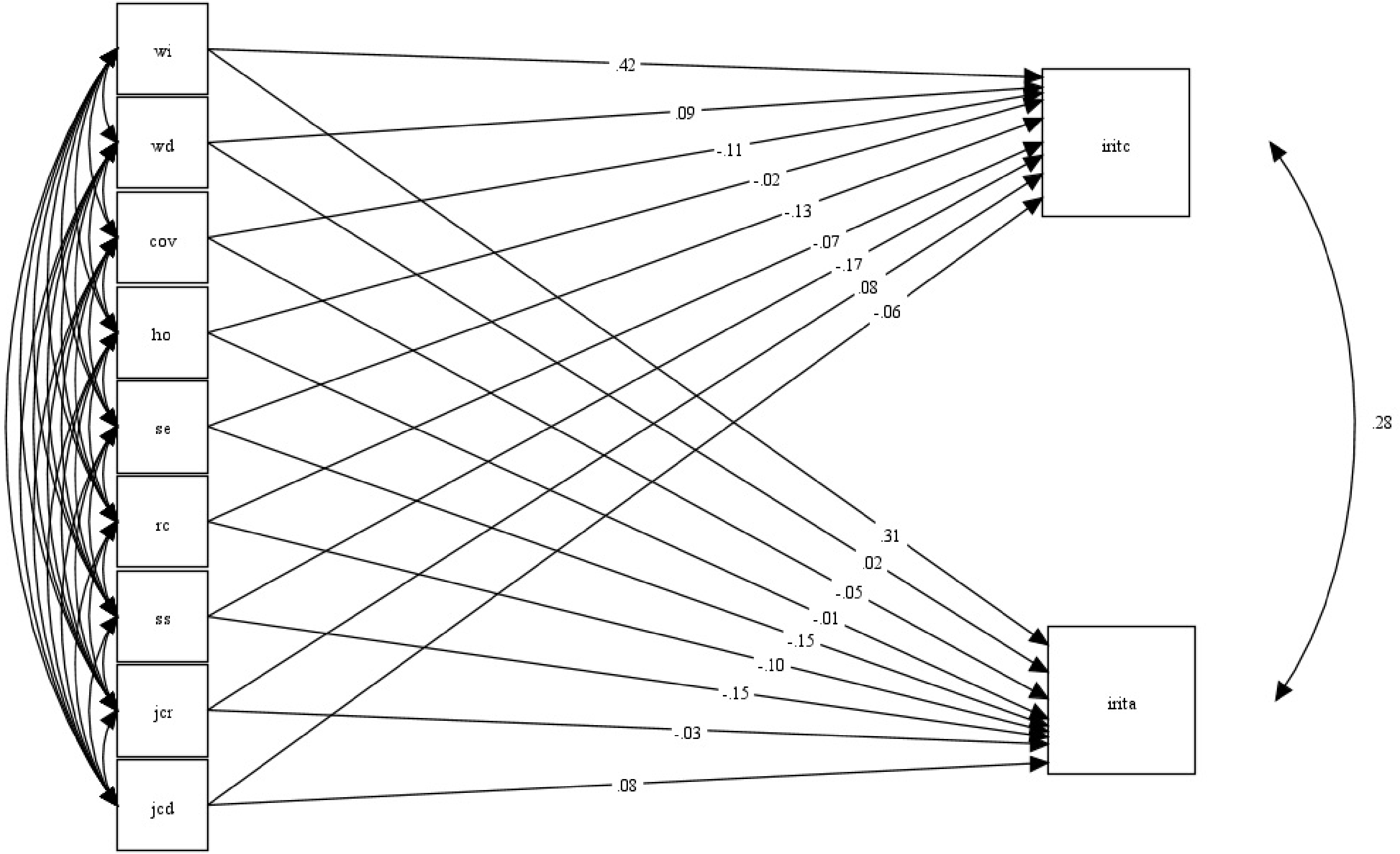

Note. iritc = cognitive irritation, irita = affective irritation, wi = work intensification, wd = change in work difficulty, cov = risk of being infected by COVID-19 during workday, ho = change in home office, se = occupational self-efficacy, rc = Emotional readiness for change, ss = social support by colleagues, jcr = job crafting – increasing resources, jcd = job crafting – hindering demands.

Supplementary Table 3

Demands and resources with moderation analyses

SYNTAX

```
ANALYSIS:
ESTIMATOR IS MLR;

MODEL:
IRITC on WI WD COV HO SE RC SS JCR JCD *interaction*;
IRITA on WI WD COV HO SE RC SS JCR JCD *interaction*;
```

Occupational self-efficacy as moderator

Occupational self-efficacy x Work intensification

MODEL RESULTS

	Estimate	S.E.	Est./S.E.	Two-Tailed P-Value
IRITC ON				
WI	0.399	0.033	12.132	0.000
WD	0.028	0.012	2.284	0.022
COV	-0.037	0.013	-2.923	0.003
HO	-0.006	0.009	-0.620	0.535
SE	-0.194	0.053	-3.663	0.000
RC	-0.083	0.047	-1.766	0.077
SS	-0.204	0.039	-5.194	0.000
JCR	0.083	0.036	2.321	0.020
JCD	-0.084	0.046	-1.804	0.071
WIXSE	-0.004	0.040	-0.108	0.914
IRITA ON				
WI	0.306	0.037	8.231	0.000
WD	0.004	0.012	0.341	0.733

COV	-0.015	0.013	-1.132	0.258
HO	-0.003	0.010	-0.251	0.802
SE	-0.211	0.058	-3.636	0.000
RC	-0.129	0.052	-2.482	0.013
SS	-0.170	0.047	-3.649	0.000
JCR	-0.030	0.040	-0.748	0.454
JCD	0.109	0.049	2.238	0.025
WIXSE	-0.108	0.048	-2.255	0.024

IRITA WITH

IRITC	0.229	0.032	7.149	0.000

Intercepts

IRITC	2.780	0.034	82.268	0.000
IRITA	2.427	0.036	67.349	0.000

Residual Variances

IRITC	0.757	0.039	19.628	0.000
IRITA	0.859	0.041	21.108	0.000

STANDARDIZED MODEL RESULTS

STDYX Standardization

				Two-Tailed
	Estimate	S.E.	Est./S.E.	P-Value

IRITC ON

WI	0.418	0.033	12.712	0.000
WD	0.085	0.037	2.285	0.022
COV	-0.108	0.037	-2.916	0.004

HO	-0.022	0.035	-0.621	0.535
SE	-0.133	0.037	-3.647	0.000
RC	-0.066	0.038	-1.765	0.078
SS	-0.171	0.033	-5.155	0.000
JCR	0.081	0.035	2.316	0.021
JCD	-0.063	0.035	-1.801	0.072
WIXSE	-0.003	0.031	-0.108	0.914
**IRITA ON**				
WI	0.321	0.038	8.426	0.000
WD	0.013	0.038	0.341	0.733
COV	-0.043	0.038	-1.132	0.258
HO	-0.009	0.037	-0.251	0.802
SE	-0.145	0.040	-3.628	0.000
RC	-0.102	0.041	-2.479	0.013
SS	-0.142	0.039	-3.653	0.000
JCR	-0.029	0.039	-0.747	0.455
JCD	0.082	0.037	2.241	0.025
WIXSE	-0.086	0.038	-2.249	0.025
**IRITA WITH**				
IRITC	0.285	0.037	7.748	0.000
**Intercepts**				
IRITC	2.681	0.064	42.115	0.000
IRITA	2.337	0.052	45.311	0.000
**Residual Variances**				
IRITC	0.704	0.029	23.870	0.000
IRITA	0.797	0.029	27.833	0.000

R-SQUARE

Observed Variable	Estimate	S.E.	Est./S.E.	Two-Tailed P-Value
IRITC	0.296	0.029	10.052	0.000
IRITA	0.203	0.029	7.104	0.000

Occupational self-efficacy x Change in work difficulty

MODEL RESULTS

	Estimate	S.E.	Est./S.E.	Two-Tailed P-Value
IRITC ON				
WI	0.399	0.033	12.125	0.000
WD	0.028	0.012	2.296	0.022
COV	-0.037	0.013	-2.945	0.003
HO	-0.006	0.009	-0.618	0.537
SE	-0.194	0.053	-3.694	0.000
RC	-0.083	0.047	-1.766	0.077
SS	-0.205	0.039	-5.201	0.000
JCR	0.083	0.036	2.321	0.020
JCD	-0.084	0.046	-1.807	0.071
WDXSE	-0.001	0.014	-0.056	0.955
IRITA ON				
WI	0.296	0.038	7.862	0.000
WD	0.006	0.013	0.484	0.629
COV	-0.016	0.013	-1.241	0.215
HO	-0.001	0.010	-0.141	0.888
SE	-0.232	0.058	-3.996	0.000

RC	-0.127	0.053	-2.391	0.017
SS	-0.178	0.047	-3.832	0.000
JCR	-0.030	0.040	-0.765	0.444
JCD	0.110	0.049	2.260	0.024
WDXSE	-0.035	0.015	-2.317	0.021

IRITA WITH

IRITC	0.230	0.032	7.130	0.000

Intercepts

IRITC	2.780	0.034	82.934	0.000
IRITA	2.432	0.036	68.173	0.000

Residual Variances

IRITC	0.757	0.039	19.618	0.000
IRITA	0.861	0.041	20.949	0.000

STANDARDIZED MODEL RESULTS

STDYX Standardization

				Two-Tailed
	Estimate	S.E.	Est./S.E.	P-Value

IRITC ON

WI	0.418	0.033	12.694	0.000
WD	0.085	0.037	2.298	0.022
COV	-0.108	0.037	-2.938	0.003
HO	-0.022	0.035	-0.618	0.537
SE	-0.134	0.036	-3.679	0.000

RC	-0.066	0.037	-1.764	0.078
SS	-0.171	0.033	-5.163	0.000
JCR	0.081	0.035	2.316	0.021
JCD	-0.063	0.035	-1.805	0.071
WDXSE	-0.002	0.032	-0.056	0.955

IRITA ON

WI	0.310	0.039	8.024	0.000
WD	0.019	0.039	0.484	0.628
COV	-0.048	0.039	-1.241	0.215
HO	-0.005	0.037	-0.141	0.888
SE	-0.160	0.040	-3.982	0.000
RC	-0.100	0.042	-2.389	0.017
SS	-0.149	0.039	-3.836	0.000
JCR	-0.029	0.038	-0.765	0.445
JCD	0.083	0.037	2.263	0.024
WDXSE	-0.078	0.034	-2.302	0.021

IRITA WITH

IRITC	0.285	0.037	7.738	0.000

Intercepts

IRITC	2.681	0.064	42.192	0.000
IRITA	2.342	0.051	46.302	0.000

Residual Variances

IRITC	0.704	0.029	23.856	0.000
IRITA	0.798	0.028	28.040	0.000

R-SQUARE

Observed			Two-Tailed	
Variable	Estimate	S.E.	Est./S.E.	P-Value

IRITC	0.296	0.029	10.046	0.000
IRITA	0.202	0.028	7.103	0.000

Occupational self-efficacy x Risk of being infected by COVID-19

MODEL RESULTS

	Estimate	S.E.	Est./S.E.	Two-Tailed P-Value
IRITC ON				
WI	0.397	0.033	11.991	0.000
WD	0.028	0.012	2.300	0.021
COV	-0.037	0.012	-3.017	0.003
HO	-0.007	0.009	-0.696	0.487
SE	-0.194	0.052	-3.704	0.000
RC	-0.086	0.047	-1.807	0.071
SS	-0.206	0.039	-5.234	0.000
JCR	0.085	0.036	2.356	0.018
JCD	-0.086	0.046	-1.874	0.061
COVXSE	0.012	0.016	0.708	0.479
IRITA ON				
WI	0.301	0.037	8.027	0.000
WD	0.005	0.013	0.382	0.703
COV	-0.015	0.013	-1.153	0.249
HO	-0.001	0.010	-0.050	0.960
SE	-0.220	0.058	-3.787	0.000
RC	-0.124	0.052	-2.360	0.018
SS	-0.174	0.047	-3.673	0.000
JCR	-0.034	0.040	-0.871	0.383

JCD	0.108	0.049	2.184	0.029
COVXSE	-0.029	0.016	-1.864	0.062

IRITA WITH

IRITC	0.231	0.032	7.180	0.000

Intercepts

IRITC	2.781	0.033	83.332	0.000
IRITA	2.436	0.036	68.361	0.000

Residual Variances

IRITC	0.756	0.038	19.648	0.000
IRITA	0.863	0.041	21.156	0.000

STANDARDIZED MODEL RESULTS

STDYX Standardization

				Two-Tailed
	Estimate	S.E.	Est./S.E.	P-Value
IRITC ON				
WI	0.416	0.033	12.572	0.000
WD	0.085	0.037	2.301	0.021
COV	-0.110	0.036	-3.008	0.003
HO	-0.024	0.035	-0.696	0.486
SE	-0.133	0.036	-3.686	0.000
RC	-0.068	0.038	-1.805	0.071
SS	-0.172	0.033	-5.194	0.000
JCR	0.082	0.035	2.350	0.019
JCD	-0.064	0.034	-1.871	0.061

COVXSE 0.025 0.035 0.707 0.480

IRITA ON

WI	0.315	0.038	8.202	0.000
WD	0.015	0.038	0.382	0.703
COV	-0.044	0.039	-1.152	0.249
HO	-0.002	0.038	-0.050	0.960
SE	-0.151	0.040	-3.777	0.000
RC	-0.098	0.042	-2.357	0.018
SS	-0.145	0.039	-3.678	0.000
JCR	-0.033	0.038	-0.871	0.384
JCD	0.081	0.037	2.185	0.029
COVXSE	-0.063	0.034	-1.858	0.063

IRITA WITH

IRITC	0.287	0.037	7.828	0.000

Intercepts

IRITC	2.682	0.064	42.215	0.000
IRITA	2.346	0.051	46.340	0.000

Residual Variances

IRITC	0.703	0.029	24.081	0.000
IRITA	0.800	0.028	28.629	0.000

R-SQUARE

Observed Variable	Estimate	S.E.	Est./S.E.	Two-Tailed P-Value
IRITC	0.297	0.029	10.170	0.000
IRITA	0.200	0.028	7.159	0.000

Occupational self-efficacy x Change in home office

MODEL RESULTS

	Estimate	S.E.	Est./S.E.	Two-Tailed P-Value
IRITC ON				
WI	0.396	0.033	11.972	0.000
WD	0.028	0.012	2.309	0.021
COV	-0.035	0.013	-2.762	0.006
HO	-0.005	0.009	-0.571	0.568
SE	-0.198	0.051	-3.847	0.000
RC	-0.081	0.047	-1.717	0.086
SS	-0.204	0.039	-5.186	0.000
JCR	0.084	0.036	2.329	0.020
JCD	-0.089	0.045	-1.969	0.049
HOXSE	-0.022	0.012	-1.822	0.068
IRITA ON				
WI	0.301	0.038	8.004	0.000
WD	0.005	0.013	0.390	0.696
COV	-0.019	0.013	-1.441	0.150
HO	-0.003	0.010	-0.289	0.773
SE	-0.215	0.058	-3.709	0.000
RC	-0.131	0.053	-2.495	0.013
SS	-0.178	0.047	-3.774	0.000
JCR	-0.031	0.040	-0.781	0.435
JCD	0.109	0.050	2.193	0.028
HOXSE	0.025	0.013	1.901	0.057
IRITA WITH				
IRITC	0.234	0.032	7.275	0.000

Intercepts

IRITC	2.780	0.033	83.570	0.000
IRITA	2.439	0.036	68.424	0.000

Residual Variances

IRITC	0.753	0.038	19.756	0.000
IRITA	0.862	0.041	21.265	0.000

STANDARDIZED MODEL RESULTS

STDYX Standardization

	Estimate	S.E.	Est./S.E.	Two-Tailed P-Value
IRITC ON				
WI	0.415	0.033	12.527	0.000
WD	0.086	0.037	2.310	0.021
COV	-0.101	0.037	-2.755	0.006
HO	-0.020	0.035	-0.572	0.568
SE	-0.136	0.036	-3.824	0.000
RC	-0.065	0.038	-1.716	0.086
SS	-0.170	0.033	-5.144	0.000
JCR	0.081	0.035	2.325	0.020
JCD	-0.067	0.034	-1.966	0.049
HOXSE	-0.060	0.033	-1.818	0.069
IRITA ON				
WI	0.315	0.038	8.185	0.000

WD	0.015	0.039	0.390	0.696
COV	-0.056	0.039	-1.441	0.150
HO	-0.011	0.037	-0.289	0.773
SE	-0.148	0.040	-3.701	0.000
RC	-0.104	0.042	-2.492	0.013
SS	-0.148	0.039	-3.781	0.000
JCR	-0.030	0.038	-0.780	0.435
JCD	0.082	0.037	2.196	0.028
HOXSE	0.068	0.036	1.902	0.057

IRITA WITH

IRITC	0.291	0.036	7.984	0.000

Intercepts

IRITC	2.681	0.063	42.309	0.000
IRITA	2.348	0.051	46.411	0.000

Residual Variances

IRITC	0.700	0.029	24.110	0.000
IRITA	0.799	0.028	28.337	0.000

R-SQUARE

Observed Variable	Estimate	S.E.	Est./S.E.	Two-Tailed P-Value
IRITC	0.300	0.029	10.323	0.000
IRITA	0.201	0.028	7.114	0.000

Emotional readiness to change as moderator

Emotional readiness to change x Work intensification

MODEL RESULTS

	Estimate	S.E.	Est./S.E.	Two-Tailed P-Value
IRITC ON				
WI	0.401	0.033	12.317	0.000
WD	0.028	0.012	2.306	0.021
COV	-0.037	0.013	-2.922	0.003
HO	-0.006	0.009	-0.602	0.547
SE	-0.194	0.052	-3.720	0.000
RC	-0.084	0.047	-1.782	0.075
SS	-0.206	0.039	-5.219	0.000
JCR	0.083	0.036	2.316	0.021
JCD	-0.088	0.047	-1.883	0.060
WIXRC	0.030	0.036	0.849	0.396
IRITA ON				
WI	0.299	0.038	7.928	0.000
WD	0.005	0.013	0.417	0.677
COV	-0.016	0.013	-1.227	0.220
HO	-0.002	0.010	-0.225	0.822
SE	-0.220	0.059	-3.707	0.000
RC	-0.129	0.053	-2.449	0.014
SS	-0.177	0.047	-3.768	0.000
JCR	-0.031	0.040	-0.774	0.439
JCD	0.101	0.049	2.066	0.039
WIXRC	0.015	0.042	0.353	0.724
IRITA WITH				
IRITC	0.229	0.032	7.107	0.000

Intercepts

IRITC	2.782	0.033	83.354	0.000
IRITA	2.439	0.036	67.674	0.000

Residual Variances

IRITC	0.756	0.038	19.635	0.000
IRITA	0.867	0.041	21.162	0.000

STANDARDIZED MODEL RESULTS

STDYX Standardization

	Estimate	S.E.	Est./S.E.	Two-Tailed P-Value
IRITC ON				
WI	0.420	0.033	12.917	0.000
WD	0.085	0.037	2.307	0.021
COV	-0.107	0.037	-2.915	0.004
HO	-0.021	0.035	-0.602	0.547
SE	-0.134	0.036	-3.706	0.000
RC	-0.067	0.037	-1.781	0.075
SS	-0.172	0.033	-5.181	0.000
JCR	0.080	0.035	2.311	0.021
JCD	-0.066	0.035	-1.881	0.060
WIXRC	0.029	0.034	0.848	0.396
IRITA ON				
WI	0.313	0.039	8.100	0.000
WD	0.016	0.039	0.417	0.676
COV	-0.047	0.038	-1.227	0.220
HO	-0.008	0.038	-0.225	0.822

SE	-0.151	0.041	-3.699	0.000
RC	-0.102	0.042	-2.446	0.014
SS	-0.148	0.039	-3.772	0.000
JCR	-0.030	0.038	-0.773	0.439
JCD	0.076	0.037	2.068	0.039
WIXRC	0.014	0.039	0.353	0.724
**IRITA WITH**				
IRITC	0.283	0.037	7.717	0.000
**Intercepts**				
IRITC	2.683	0.063	42.336	0.000
IRITA	2.349	0.050	46.598	0.000
**Residual Variances**				
IRITC	0.703	0.029	23.896	0.000
IRITA	0.804	0.028	28.895	0.000

Emotional readiness to change x Change in work difficulty

MODEL RESULTS

				Two-Tailed
	Estimate	S.E.	Est./S.E.	P-Value
**IRITC ON**				
WI	0.398	0.033	12.067	0.000
WD	0.028	0.012	2.289	0.022
COV	-0.037	0.013	-2.974	0.003
HO	-0.006	0.009	-0.625	0.532
SE	-0.194	0.052	-3.702	0.000
RC	-0.084	0.047	-1.779	0.075

SS	-0.204	0.039	-5.198	0.000
JCR	0.084	0.036	2.327	0.020
JCD	-0.083	0.046	-1.793	0.073
WDXRC	-0.004	0.012	-0.287	0.774

IRITA ON

WI	0.301	0.039	7.815	0.000
WD	0.005	0.013	0.405	0.686
COV	-0.016	0.013	-1.177	0.239
HO	-0.002	0.010	-0.212	0.832
SE	-0.219	0.060	-3.651	0.000
RC	-0.128	0.053	-2.408	0.016
SS	-0.178	0.047	-3.798	0.000
JCR	-0.031	0.040	-0.789	0.430
JCD	0.099	0.049	2.004	0.045
WDXRC	0.018	0.014	1.275	0.202

IRITA WITH

IRITC	0.230	0.032	7.120	0.000

Intercepts

IRITC	2.779	0.034	82.820	0.000
IRITA	2.443	0.036	67.422	0.000

Residual Variances

IRITC	0.757	0.039	19.601	0.000
IRITA	0.865	0.041	21.053	0.000

STANDARDIZED MODEL RESULTS

STDYX Standardization

	Estimate	S.E.	Est./S.E.	Two-Tailed P-Value
IRITC ON				
WI	0.417	0.033	12.638	0.000
WD	0.085	0.037	2.291	0.022
COV	-0.109	0.037	-2.966	0.003
HO	-0.022	0.035	-0.626	0.532
SE	-0.134	0.036	-3.685	0.000
RC	-0.066	0.037	-1.778	0.075
SS	-0.171	0.033	-5.161	0.000
JCR	0.081	0.035	2.322	0.020
JCD	-0.062	0.035	-1.791	0.073
WDXRC	-0.009	0.033	-0.287	0.774
IRITA ON				
WI	0.315	0.039	7.979	0.000
WD	0.016	0.039	0.405	0.686
COV	-0.045	0.039	-1.177	0.239
HO	-0.008	0.038	-0.212	0.832
SE	-0.150	0.041	-3.645	0.000
RC	-0.101	0.042	-2.406	0.016
SS	-0.149	0.039	-3.801	0.000
JCR	-0.030	0.039	-0.788	0.430
JCD	0.074	0.037	2.005	0.045
WDXRC	0.046	0.036	1.274	0.203
IRITA WITH				
IRITC	0.285	0.037	7.767	0.000

Intercepts

IRITC	2.680	0.063	42.299	0.000
IRITA	2.353	0.050	46.682	0.000

Residual Variances

IRITC	0.704	0.030	23.846	0.000
IRITA	0.802	0.028	28.442	0.000

R-SQUARE

Observed Variable	Estimate	S.E.	Two-Tailed Est./S.E.	P-Value
IRITC	0.296	0.030	10.045	0.000
IRITA	0.198	0.028	7.034	0.000

Emotional readiness to change x Risk of being infected by COVID-19

MODEL RESULTS

	Estimate	S.E.	Two-Tailed Est./S.E.	P-Value
IRITC ON				
WI	0.401	0.033	12.274	0.000
WD	0.028	0.012	2.303	0.021
COV	-0.036	0.013	-2.885	0.004
HO	-0.007	0.009	-0.750	0.453
SE	-0.198	0.052	-3.803	0.000
RC	-0.085	0.047	-1.819	0.069
SS	-0.204	0.039	-5.190	0.000
JCR	0.085	0.036	2.360	0.018
JCD	-0.087	0.046	-1.916	0.055
COVXRC	0.024	0.014	1.683	0.092

IRITA ON

WI	0.301	0.037	8.051	0.000
WD	0.005	0.013	0.426	0.670
COV	-0.015	0.013	-1.175	0.240
HO	-0.004	0.010	-0.384	0.701
SE	-0.225	0.059	-3.817	0.000
RC	-0.131	0.052	-2.536	0.011
SS	-0.176	0.047	-3.757	0.000
JCR	-0.029	0.040	-0.731	0.465
JCD	0.099	0.049	2.020	0.043
COVXRC	0.029	0.014	2.076	0.038

IRITA WITH

IRITC	0.225	0.032	6.994	0.000

Intercepts

IRITC	2.788	0.033	83.539	0.000
IRITA	2.448	0.036	67.862	0.000

Residual Variances

IRITC	0.753	0.038	19.698	0.000
IRITA	0.862	0.041	21.128	0.000

STANDARDIZED MODEL RESULTS

STDYX Standardization

				Two-Tailed
	Estimate	S.E.	Est./S.E.	P-Value

IRITC ON

WI	0.421	0.033	12.863	0.000
WD	0.085	0.037	2.304	0.021
COV	-0.106	0.037	-2.879	0.004
HO	-0.026	0.035	-0.751	0.453
SE	-0.136	0.036	-3.787	0.000
RC	-0.068	0.037	-1.816	0.069
SS	-0.170	0.033	-5.151	0.000
JCR	0.082	0.035	2.355	0.019
JCD	-0.066	0.034	-1.913	0.056
COVXRC	0.060	0.036	1.676	0.094

IRITA ON

WI	0.315	0.038	8.217	0.000
WD	0.016	0.039	0.427	0.670
COV	-0.045	0.038	-1.176	0.240
HO	-0.014	0.038	-0.384	0.701
SE	-0.155	0.041	-3.812	0.000
RC	-0.104	0.041	-2.532	0.011
SS	-0.146	0.039	-3.759	0.000
JCR	-0.028	0.038	-0.730	0.465
JCD	0.074	0.037	2.023	0.043
COVXRC	0.071	0.035	2.064	0.039

IRITA WITH

IRITC	0.280	0.037	7.583	0.000

Intercepts

IRITC	2.689	0.064	42.191	0.000
IRITA	2.357	0.051	46.406	0.000

Residual Variances

IRITC	0.700	0.029	24.131	0.000
IRITA	0.799	0.028	28.792	0.000

R-SQUARE

Observed Variable	Estimate	S.E.	Est./S.E.	Two-Tailed P-Value
IRITC	0.300	0.029	10.332	0.000
IRITA	0.201	0.028	7.250	0.000

Emotional readiness to change x Change in home office

MODEL RESULTS

	Estimate	S.E.	Est./S.E.	Two-Tailed P-Value
IRITC ON				
WI	0.399	0.033	12.126	0.000
WD	0.027	0.012	2.271	0.023
COV	-0.037	0.013	-2.911	0.004
HO	-0.006	0.009	-0.658	0.511
SE	-0.193	0.052	-3.695	0.000
RC	-0.085	0.047	-1.802	0.072
SS	-0.205	0.039	-5.205	0.000
JCR	0.083	0.036	2.323	0.020
JCD	-0.084	0.046	-1.827	0.068
HOXRC	-0.004	0.011	-0.413	0.680
IRITA ON				
WI	0.298	0.038	7.832	0.000

WD	0.005	0.013	0.434	0.665
COV	-0.017	0.013	-1.266	0.205
HO	-0.002	0.010	-0.181	0.856
SE	-0.221	0.059	-3.722	0.000
RC	-0.127	0.053	-2.403	0.016
SS	-0.176	0.047	-3.756	0.000
JCR	-0.031	0.040	-0.770	0.441
JCD	0.104	0.049	2.102	0.036
HOXRC	0.007	0.012	0.562	0.574

IRITA WITH

IRITC	0.230	0.032	7.109	0.000

Intercepts

IRITC	2.782	0.033	83.457	0.000
IRITA	2.435	0.036	68.411	0.000

Residual Variances

IRITC	0.757	0.039	19.599	0.000
IRITA	0.866	0.041	21.198	0.000

STANDARDIZED MODEL RESULTS

STDYX Standardization

				Two-Tailed
	Estimate	S.E.	Est./S.E.	P-Value

IRITC ON

WI	0.418	0.033	12.699	0.000
WD	0.084	0.037	2.272	0.023

COV	-0.108	0.037	-2.904	0.004
HO	-0.023	0.035	-0.658	0.510
SE	-0.133	0.036	-3.679	0.000
RC	-0.067	0.037	-1.800	0.072
SS	-0.171	0.033	-5.166	0.000
JCR	0.081	0.035	2.318	0.020
JCD	-0.063	0.035	-1.824	0.068
HOXRC	-0.014	0.033	-0.413	0.680

IRITA ON

WI	0.312	0.039	8.002	0.000
WD	0.017	0.039	0.434	0.664
COV	-0.049	0.039	-1.266	0.206
HO	-0.007	0.038	-0.181	0.856
SE	-0.152	0.041	-3.714	0.000
RC	-0.100	0.042	-2.401	0.016
SS	-0.147	0.039	-3.760	0.000
JCR	-0.030	0.039	-0.769	0.442
JCD	0.077	0.037	2.104	0.035
HOXRC	0.021	0.037	0.562	0.574

IRITA WITH

IRITC	0.284	0.037	7.744	0.000

Intercepts

IRITC	2.683	0.064	42.103	0.000
IRITA	2.345	0.051	46.321	0.000

Residual Variances

IRITC	0.704	0.029	23.935	0.000
IRITA	0.803	0.028	28.745	0.000

R-SQUARE

Observed Variable	Estimate	S.E.	Est./S.E.	Two-Tailed P-Value
IRITC	0.296	0.029	10.088	0.000
IRITA	0.197	0.028	7.033	0.000

Social support by colleagues as moderator

Social support by colleagues x Work intensification

MODEL RESULTS

	Estimate	S.E.	Est./S.E.	Two-Tailed P-Value
IRITC ON				
WI	0.398	0.033	12.131	0.000
WD	0.028	0.012	2.312	0.021
COV	-0.037	0.013	-2.938	0.003
HO	-0.006	0.009	-0.600	0.549
SE	-0.192	0.052	-3.672	0.000
RC	-0.082	0.047	-1.734	0.083
SS	-0.205	0.039	-5.257	0.000
JCR	0.083	0.036	2.313	0.021
JCD	-0.082	0.046	-1.772	0.076
WIXSS	-0.025	0.033	-0.762	0.446
IRITA ON				
WI	0.298	0.038	7.871	0.000
WD	0.005	0.013	0.429	0.668
COV	-0.016	0.013	-1.234	0.217
HO	-0.002	0.010	-0.221	0.825

SE	-0.219	0.059	-3.675	0.000
RC	-0.128	0.053	-2.413	0.016
SS	-0.177	0.047	-3.767	0.000
JCR	-0.031	0.040	-0.774	0.439
JCD	0.104	0.049	2.119	0.034
WIXSS	-0.016	0.041	-0.390	0.697

IRITA WITH

IRITC	0.229	0.032	7.064	0.000

Intercepts

IRITC	2.779	0.033	83.128	0.000
IRITA	2.438	0.036	68.071	0.000

Residual Variances

IRITC	0.756	0.039	19.577	0.000
IRITA	0.867	0.041	21.146	0.000

STANDARDIZED MODEL RESULTS

STDYX Standardization

				Two-Tailed
	Estimate	S.E.	Est./S.E.	P-Value

IRITC ON

WI	0.417	0.033	12.702	0.000
WD	0.086	0.037	2.314	0.021
COV	-0.108	0.037	-2.932	0.003
HO	-0.021	0.035	-0.600	0.548

SE	-0.132	0.036	-3.655	0.000
RC	-0.065	0.038	-1.732	0.083
SS	-0.172	0.033	-5.218	0.000
JCR	0.081	0.035	2.308	0.021
JCD	-0.061	0.035	-1.770	0.077
WIXSS	-0.024	0.032	-0.762	0.446

IRITA ON

WI	0.312	0.039	8.037	0.000
WD	0.017	0.039	0.429	0.668
COV	-0.048	0.039	-1.234	0.217
HO	-0.008	0.038	-0.221	0.825
SE	-0.150	0.041	-3.666	0.000
RC	-0.102	0.042	-2.410	0.016
SS	-0.147	0.039	-3.771	0.000
JCR	-0.030	0.039	-0.773	0.440
JCD	0.078	0.037	2.121	0.034
WIXSS	-0.015	0.039	-0.390	0.697

IRITA WITH

IRITC	0.283	0.037	7.691	0.000

Intercepts

IRITC	2.680	0.064	42.150	0.000
IRITA	2.347	0.050	46.534	0.000

Residual Variances

IRITC	0.703	0.030	23.652	0.000
IRITA	0.804	0.028	28.674	0.000

R-SQUARE

Observed Two-Tailed

Variable	Estimate	S.E.	Est./S.E.	P-Value
IRITC	0.297	0.030	9.987	0.000
IRITA	0.196	0.028	7.007	0.000

Social support by colleagues x Change in work difficulty

MODEL RESULTS

				Two-Tailed
	Estimate	S.E.	Est./S.E.	P-Value
IRITC ON				
WI	0.399	0.033	12.081	0.000
WD	0.028	0.012	2.285	0.022
COV	-0.037	0.013	-2.950	0.003
HO	-0.006	0.009	-0.622	0.534
SE	-0.194	0.052	-3.698	0.000
RC	-0.083	0.047	-1.768	0.077
SS	-0.205	0.040	-5.126	0.000
JCR	0.083	0.036	2.322	0.020
JCD	-0.084	0.046	-1.823	0.068
WDXSS	0.000	0.012	0.026	0.979
IRITA ON				
WI	0.298	0.038	7.863	0.000
WD	0.005	0.013	0.404	0.687
COV	-0.016	0.013	-1.230	0.219
HO	-0.002	0.010	-0.221	0.825
SE	-0.220	0.059	-3.703	0.000
RC	-0.129	0.053	-2.419	0.016
SS	-0.176	0.047	-3.752	0.000

JCR	-0.031	0.040	-0.770	0.441
JCD	0.104	0.049	2.112	0.035
WDXSS	-0.003	0.013	-0.198	0.843
**IRITA WITH**				
IRITC	0.230	0.032	7.084	0.000
**Intercepts**				
IRITC	2.780	0.034	82.779	0.000
IRITA	2.438	0.036	68.061	0.000
**Residual Variances**				
IRITC	0.757	0.039	19.616	0.000
IRITA	0.867	0.041	21.143	0.000

STANDARDIZED MODEL RESULTS

STDYX Standardization

	Estimate	S.E.	Est./S.E.	Two-Tailed P-Value
**IRITC ON**				
WI	0.418	0.033	12.645	0.000
WD	0.085	0.037	2.286	0.022
COV	-0.108	0.037	-2.942	0.003
HO	-0.022	0.035	-0.622	0.534
SE	-0.134	0.036	-3.682	0.000
RC	-0.066	0.038	-1.766	0.077
SS	-0.171	0.034	-5.091	0.000
JCR	0.081	0.035	2.318	0.020

JCD	-0.063	0.035	-1.820	0.069
WDXSS	0.001	0.034	0.026	0.979

IRITA ON

WI	0.312	0.039	8.033	0.000
WD	0.016	0.039	0.404	0.686
COV	-0.047	0.039	-1.229	0.219
HO	-0.008	0.038	-0.221	0.825
SE	-0.151	0.041	-3.696	0.000
RC	-0.102	0.042	-2.416	0.016
SS	-0.146	0.039	-3.755	0.000
JCR	-0.030	0.039	-0.770	0.442
JCD	0.078	0.037	2.114	0.034
WDXSS	-0.007	0.037	-0.198	0.843

IRITA WITH

IRITC	0.284	0.037	7.707	0.000

Intercepts

IRITC	2.681	0.063	42.269	0.000
IRITA	2.347	0.050	46.565	0.000

Residual Variances

IRITC	0.704	0.029	23.860	0.000
IRITA	0.804	0.028	28.791	0.000

R-SQUARE

Observed Variable	Estimate	S.E.	Est./S.E.	Two-Tailed P-Value
IRITC	0.296	0.029	10.047	0.000

IRITA	0.196	0.028	7.028	0.000

Social support by colleagues x Risk of being infected by COVID-19

MODEL RESULTS

				Two-Tailed
	Estimate	S.E.	Est./S.E.	P-Value
IRITC ON				
WI	0.399	0.033	12.107	0.000
WD	0.028	0.012	2.283	0.022
COV	-0.037	0.013	-2.921	0.003
HO	-0.006	0.009	-0.620	0.535
SE	-0.194	0.052	-3.705	0.000
RC	-0.083	0.047	-1.763	0.078
SS	-0.205	0.039	-5.258	0.000
JCR	0.083	0.036	2.323	0.020
JCD	-0.084	0.046	-1.821	0.069
COVXSS	0.002	0.013	0.134	0.893
IRITA ON				
WI	0.298	0.038	7.851	0.000
WD	0.005	0.013	0.406	0.685
COV	-0.016	0.013	-1.233	0.218
HO	-0.002	0.010	-0.234	0.815
SE	-0.220	0.060	-3.688	0.000
RC	-0.129	0.053	-2.427	0.015
SS	-0.177	0.047	-3.786	0.000
JCR	-0.030	0.040	-0.765	0.444
JCD	0.103	0.049	2.090	0.037
COVXSS	0.002	0.015	0.139	0.889

IRITA WITH				
IRITC	0.230	0.032	7.081	0.000
Intercepts				
IRITC	2.781	0.033	83.017	0.000
IRITA	2.439	0.036	67.839	0.000
Residual Variances				
IRITC	0.757	0.039	19.615	0.000
IRITA	0.867	0.041	21.135	0.000

STANDARDIZED MODEL RESULTS

STDYX Standardization

	Estimate	S.E.	Est./S.E.	Two-Tailed P-Value
IRITC ON				
WI	0.418	0.033	12.679	0.000
WD	0.085	0.037	2.284	0.022
COV	-0.108	0.037	-2.913	0.004
HO	-0.022	0.035	-0.620	0.535
SE	-0.134	0.036	-3.689	0.000
RC	-0.066	0.038	-1.762	0.078
SS	-0.171	0.033	-5.218	0.000
JCR	0.081	0.035	2.318	0.020
JCD	-0.063	0.035	-1.819	0.069
COVXSS	0.005	0.035	0.134	0.893

IRITA ON

WI	0.312	0.039	8.017	0.000
WD	0.016	0.039	0.406	0.685
COV	-0.047	0.038	-1.232	0.218
HO	-0.009	0.038	-0.234	0.815
SE	-0.151	0.041	-3.680	0.000
RC	-0.102	0.042	-2.424	0.015
SS	-0.148	0.039	-3.789	0.000
JCR	-0.030	0.039	-0.765	0.444
JCD	0.077	0.037	2.092	0.036
COVXSS	0.005	0.039	0.139	0.889

IRITA WITH

IRITC	0.284	0.037	7.705	0.000

Intercepts

IRITC	2.682	0.063	42.261	0.000
IRITA	2.348	0.050	46.639	0.000

Residual Variances

IRITC	0.704	0.029	23.861	0.000
IRITA	0.804	0.028	28.811	0.000

R-SQUARE

Observed Variable	Estimate	S.E.	Two-Tailed Est./S.E.	P-Value
IRITC	0.296	0.029	10.049	0.000
IRITA	0.196	0.028	7.032	0.000

Social support by colleagues x Change in home office

MODEL RESULTS

	Estimate	S.E.	Est./S.E.	Two-Tailed P-Value
IRITC ON				
WI	0.399	0.033	12.112	0.000
WD	0.029	0.012	2.362	0.018
COV	-0.037	0.012	-2.968	0.003
HO	-0.007	0.009	-0.707	0.480
SE	-0.192	0.052	-3.697	0.000
RC	-0.086	0.047	-1.815	0.069
SS	-0.209	0.039	-5.308	0.000
JCR	0.086	0.036	2.396	0.017
JCD	-0.088	0.046	-1.906	0.057
HOXSS	-0.020	0.009	-2.107	0.035
IRITA ON				
WI	0.298	0.038	7.900	0.000
WD	0.007	0.013	0.517	0.605
COV	-0.016	0.013	-1.251	0.211
HO	-0.003	0.010	-0.343	0.731
SE	-0.217	0.058	-3.719	0.000
RC	-0.132	0.052	-2.519	0.012
SS	-0.183	0.047	-3.920	0.000
JCR	-0.027	0.040	-0.676	0.499
JCD	0.097	0.049	1.998	0.046
HOXSS	-0.028	0.011	-2.667	0.008
IRITA WITH				
IRITC	0.223	0.033	6.844	0.000

Intercepts

IRITC	2.786	0.034	83.046	0.000
IRITA	2.446	0.036	68.397	0.000

Residual Variances

IRITC	0.752	0.038	19.633	0.000
IRITA	0.857	0.041	21.086	0.000

STANDARDIZED MODEL RESULTS

STDYX Standardization

	Estimate	S.E.	Est./S.E.	Two-Tailed P-Value
IRITC ON				
WI	0.418	0.033	12.667	0.000
WD	0.088	0.037	2.364	0.018
COV	-0.108	0.037	-2.962	0.003
HO	-0.025	0.035	-0.708	0.479
SE	-0.132	0.036	-3.679	0.000
RC	-0.068	0.037	-1.814	0.070
SS	-0.175	0.033	-5.262	0.000
JCR	0.083	0.035	2.391	0.017
JCD	-0.066	0.035	-1.903	0.057
HOXSS	-0.066	0.031	-2.099	0.036
IRITA ON				
WI	0.312	0.039	8.061	0.000
WD	0.020	0.039	0.517	0.605

COV	-0.048	0.038	-1.252	0.211
HO	-0.013	0.038	-0.343	0.731
SE	-0.149	0.040	-3.710	0.000
RC	-0.105	0.042	-2.516	0.012
SS	-0.153	0.039	-3.914	0.000
JCR	-0.026	0.038	-0.676	0.499
JCD	0.073	0.036	2.000	0.046
HOXSS	-0.094	0.035	-2.659	0.008

IRITA WITH

IRITC	0.278	0.037	7.443	0.000

Intercepts

IRITC	2.687	0.063	42.310	0.000
IRITA	2.356	0.050	46.870	0.000

Residual Variances

IRITC	0.699	0.029	23.925	0.000
IRITA	0.795	0.028	28.196	0.000

R-SQUARE

Observed Variable	Estimate	S.E.	Est./S.E.	Two-Tailed P-Value
IRITC	0.301	0.029	10.282	0.000
IRITA	0.205	0.028	7.268	0.000

Job crafting as moderator

Job crafting - increasing resources x Work intensification

MODEL RESULTS

			Two-Tailed	
	Estimate	S.E.	Est./S.E.	P-Value
**IRITC ON**				
WI	0.400	0.033	12.164	0.000
WD	0.028	0.012	2.291	0.022
COV	-0.037	0.013	-2.954	0.003
HO	-0.006	0.009	-0.642	0.521
SE	-0.195	0.052	-3.711	0.000
RC	-0.084	0.047	-1.778	0.075
SS	-0.204	0.039	-5.206	0.000
JCR	0.083	0.036	2.310	0.021
JCD	-0.086	0.047	-1.854	0.064
WIXJCR	0.016	0.028	0.566	0.572
**IRITA ON**				
WI	0.297	0.038	7.815	0.000
WD	0.005	0.013	0.410	0.682
COV	-0.016	0.013	-1.226	0.220
HO	-0.002	0.010	-0.213	0.832
SE	-0.219	0.060	-3.680	0.000
RC	-0.129	0.053	-2.423	0.015
SS	-0.177	0.047	-3.768	0.000
JCR	-0.030	0.040	-0.756	0.449
JCD	0.106	0.049	2.165	0.030
WIXJCR	-0.016	0.033	-0.500	0.617
**IRITA WITH**				
IRITC	0.230	0.032	7.095	0.000
**Intercepts**				
IRITC	2.779	0.034	82.924	0.000

IRITA	2.439	0.036	68.261	0.000

Residual Variances

IRITC	0.756	0.039	19.609	0.000
IRITA	0.867	0.041	21.140	0.000

STANDARDIZED MODEL RESULTS

STDYX Standardization

	Estimate	S.E.	Est./S.E.	Two-Tailed P-Value
IRITC ON				
WI	0.419	0.033	12.754	0.000
WD	0.085	0.037	2.293	0.022
COV	-0.109	0.037	-2.946	0.003
HO	-0.022	0.035	-0.643	0.520
SE	-0.134	0.036	-3.695	0.000
RC	-0.067	0.037	-1.776	0.076
SS	-0.171	0.033	-5.168	0.000
JCR	0.080	0.035	2.305	0.021
JCD	-0.065	0.035	-1.852	0.064
WIXJCR	0.018	0.031	0.566	0.571
IRITA ON				
WI	0.310	0.039	7.977	0.000
WD	0.016	0.039	0.410	0.682
COV	-0.047	0.039	-1.226	0.220
HO	-0.008	0.038	-0.213	0.832

SE	-0.151	0.041	-3.671	0.000
RC	-0.102	0.042	-2.420	0.016
SS	-0.147	0.039	-3.772	0.000
JCR	-0.029	0.038	-0.756	0.450
JCD	0.079	0.037	2.168	0.030
WIXJCR	-0.018	0.037	-0.500	0.617

IRITA WITH

IRITC	0.284	0.037	7.714	0.000

Intercepts

IRITC	2.680	0.063	42.242	0.000
IRITA	2.349	0.050	46.805	0.000

Residual Variances

IRITC	0.703	0.030	23.768	0.000
IRITA	0.804	0.028	28.722	0.000

R-SQUARE

Observed			Two-Tailed	
Variable	Estimate	S.E.	Est./S.E.	P-Value
IRITC	0.297	0.030	10.023	0.000
IRITA	0.196	0.028	7.023	0.000

Job crafting - increasing resources x Change in work difficulty

MODEL RESULTS

			Two-Tailed	
	Estimate	S.E.	Est./S.E.	P-Value

IRITC ON

WI	0.397	0.033	12.081	0.000
WD	0.028	0.012	2.328	0.020
COV	-0.037	0.013	-2.933	0.003
HO	-0.006	0.009	-0.623	0.533
SE	-0.194	0.052	-3.718	0.000
RC	-0.082	0.047	-1.745	0.081
SS	-0.204	0.040	-5.128	0.000
JCR	0.084	0.036	2.332	0.020
JCD	-0.079	0.046	-1.714	0.087
WDXJCR	-0.015	0.010	-1.408	0.159

IRITA ON

WI	0.299	0.038	7.849	0.000
WD	0.005	0.013	0.402	0.688
COV	-0.016	0.013	-1.244	0.213
HO	-0.002	0.010	-0.232	0.816
SE	-0.220	0.060	-3.690	0.000
RC	-0.130	0.053	-2.435	0.015
SS	-0.177	0.047	-3.772	0.000
JCR	-0.031	0.040	-0.768	0.443
JCD	0.102	0.049	2.062	0.039
WDXJCR	0.005	0.011	0.465	0.642

IRITA WITH

IRITC	0.231	0.032	7.142	0.000

Intercepts

IRITC	2.780	0.033	83.436	0.000
IRITA	2.438	0.036	68.253	0.000

Residual Variances

IRITC	0.754	0.039	19.571	0.000

IRITA	0.867	0.041	21.097	0.000

STANDARDIZED MODEL RESULTS

STDYX Standardization

	Estimate	S.E.	Est./S.E.	Two-Tailed P-Value
IRITC ON				
WI	0.416	0.033	12.645	0.000
WD	0.086	0.037	2.330	0.020
COV	-0.108	0.037	-2.927	0.003
HO	-0.022	0.035	-0.624	0.533
SE	-0.134	0.036	-3.701	0.000
RC	-0.065	0.037	-1.743	0.081
SS	-0.170	0.033	-5.090	0.000
JCR	0.081	0.035	2.328	0.020
JCD	-0.059	0.035	-1.712	0.087
WDXJCR	-0.047	0.033	-1.405	0.160
IRITA ON				
WI	0.312	0.039	8.016	0.000
WD	0.016	0.039	0.402	0.688
COV	-0.048	0.039	-1.244	0.213
HO	-0.009	0.038	-0.232	0.816
SE	-0.151	0.041	-3.682	0.000
RC	-0.103	0.042	-2.432	0.015
SS	-0.147	0.039	-3.775	0.000
JCR	-0.030	0.039	-0.767	0.443
JCD	0.076	0.037	2.064	0.039

WDXJCR	0.016	0.035	0.465	0.642

IRITA WITH

IRITC	0.285	0.037	7.796	0.000

Intercepts

IRITC	2.681	0.063	42.223	0.000
IRITA	2.348	0.050	46.701	0.000

Residual Variances

IRITC	0.702	0.029	23.959	0.000
IRITA	0.804	0.028	28.725	0.000

R-SQUARE

Observed			Two-Tailed	
Variable	Estimate	S.E.	Est./S.E.	P-Value
IRITC	0.298	0.029	10.194	0.000
IRITA	0.196	0.028	7.021	0.000

Job crafting - increasing resources x Risk of being infected by COVID-19

MODEL RESULTS

			Two-Tailed	
	Estimate	S.E.	Est./S.E.	P-Value
IRITC ON				
WI	0.399	0.033	12.115	0.000
WD	0.027	0.012	2.278	0.023
COV	-0.037	0.013	-2.943	0.003

HO	-0.006	0.009	-0.612	0.541
SE	-0.193	0.052	-3.695	0.000
RC	-0.084	0.047	-1.767	0.077
SS	-0.205	0.039	-5.205	0.000
JCR	0.084	0.036	2.324	0.020
JCD	-0.085	0.046	-1.847	0.065
COVXJCR	0.004	0.012	0.332	0.740

IRITA ON

WI	0.298	0.038	7.888	0.000
WD	0.005	0.013	0.398	0.691
COV	-0.016	0.013	-1.231	0.218
HO	-0.002	0.010	-0.218	0.827
SE	-0.219	0.059	-3.697	0.000
RC	-0.129	0.053	-2.436	0.015
SS	-0.177	0.047	-3.766	0.000
JCR	-0.030	0.040	-0.754	0.451
JCD	0.100	0.049	2.026	0.043
COVXJCR	0.008	0.011	0.724	0.469

IRITA WITH

IRITC	0.229	0.032	7.075	0.000

Intercepts

IRITC	2.781	0.033	83.330	0.000
IRITA	2.439	0.036	68.344	0.000

Residual Variances

IRITC	0.757	0.039	19.627	0.000
IRITA	0.866	0.041	21.113	0.000

STANDARDIZED MODEL RESULTS

STDYX Standardization

	Estimate	S.E.	Est./S.E.	Two-Tailed P-Value
IRITC ON				
WI	0.418	0.033	12.688	0.000
WD	0.085	0.037	2.279	0.023
COV	-0.108	0.037	-2.936	0.003
HO	-0.021	0.035	-0.613	0.540
SE	-0.133	0.036	-3.679	0.000
RC	-0.066	0.038	-1.766	0.077
SS	-0.171	0.033	-5.167	0.000
JCR	0.081	0.035	2.320	0.020
JCD	-0.064	0.035	-1.844	0.065
COVXJCR	0.012	0.036	0.332	0.740
IRITA ON				
WI	0.312	0.039	8.057	0.000
WD	0.015	0.039	0.398	0.691
COV	-0.048	0.039	-1.231	0.218
HO	-0.008	0.038	-0.218	0.827
SE	-0.151	0.041	-3.690	0.000
RC	-0.102	0.042	-2.433	0.015
SS	-0.148	0.039	-3.770	0.000
JCR	-0.029	0.039	-0.754	0.451
JCD	0.075	0.037	2.028	0.043
COVXJCR	0.025	0.034	0.724	0.469

IRITA WITH

IRITC	0.283	0.037	7.696	0.000

Intercepts

IRITC	2.681	0.063	42.326	0.000
IRITA	2.349	0.050	46.727	0.000

Residual Variances

IRITC	0.704	0.029	23.867	0.000
IRITA	0.803	0.028	28.703	0.000

R-SQUARE

Observed Variable	Estimate	S.E.	Two-Tailed Est./S.E.	P-Value
IRITC	0.296	0.029	10.057	0.000
IRITA	0.197	0.028	7.031	0.000

Job crafting - increasing resources x Change in home office

MODEL RESULTS

	Estimate	S.E.	Two-Tailed Est./S.E.	P-Value
IRITC ON				
WI	0.400	0.033	12.223	0.000
WD	0.027	0.012	2.267	0.023
COV	-0.037	0.013	-2.984	0.003
HO	-0.005	0.009	-0.583	0.560
SE	-0.193	0.052	-3.703	0.000
RC	-0.085	0.047	-1.789	0.074
SS	-0.203	0.039	-5.176	0.000

JCR	0.079	0.036	2.214	0.027
JCD	-0.083	0.046	-1.805	0.071
HOXJCR	-0.016	0.009	-1.811	0.070

IRITA ON

WI	0.300	0.037	8.010	0.000
WD	0.005	0.013	0.382	0.702
COV	-0.017	0.013	-1.269	0.205
HO	-0.002	0.010	-0.198	0.843
SE	-0.219	0.059	-3.715	0.000
RC	-0.130	0.053	-2.470	0.014
SS	-0.174	0.046	-3.751	0.000
JCR	-0.035	0.040	-0.877	0.380
JCD	0.104	0.049	2.115	0.034
HOXJCR	-0.017	0.009	-1.789	0.074

IRITA WITH

IRITC	0.226	0.032	7.051	0.000

Intercepts

IRITC	2.781	0.033	83.544	0.000
IRITA	2.438	0.036	68.409	0.000

Residual Variances

IRITC	0.753	0.038	19.820	0.000
IRITA	0.863	0.041	21.176	0.000

STANDARDIZED MODEL RESULTS

STDYX Standardization

	Estimate	S.E.	Est./S.E.	Two-Tailed P-Value
IRITC ON				
WI	0.419	0.033	12.814	0.000
WD	0.084	0.037	2.268	0.023
COV	-0.110	0.037	-2.976	0.003
HO	-0.020	0.035	-0.584	0.559
SE	-0.133	0.036	-3.686	0.000
RC	-0.067	0.038	-1.788	0.074
SS	-0.169	0.033	-5.136	0.000
JCR	0.077	0.035	2.210	0.027
JCD	-0.062	0.035	-1.802	0.072
HOXJCR	-0.060	0.033	-1.814	0.070
IRITA ON				
WI	0.314	0.038	8.188	0.000
WD	0.015	0.039	0.382	0.702
COV	-0.049	0.039	-1.268	0.205
HO	-0.007	0.038	-0.198	0.843
SE	-0.150	0.041	-3.707	0.000
RC	-0.103	0.042	-2.467	0.014
SS	-0.145	0.039	-3.754	0.000
JCR	-0.034	0.039	-0.877	0.381
JCD	0.078	0.037	2.117	0.034
HOXJCR	-0.063	0.035	-1.792	0.073
IRITA WITH				
IRITC	0.280	0.037	7.628	0.000
Intercepts				
IRITC	2.681	0.063	42.371	0.000

IRITA	2.348	0.050	46.652	0.000

Residual Variances

IRITC	0.700	0.029	23.984	0.000
IRITA	0.800	0.028	28.320	0.000

R-SQUARE

Observed			Two-Tailed	
Variable	Estimate	S.E.	Est./S.E.	P-Value
IRITC	0.300	0.029	10.271	0.000
IRITA	0.200	0.028	7.083	0.000

Job crafting - hindering demands x Work intensification

MODEL RESULTS

			Two-Tailed	
	Estimate	S.E.	Est./S.E.	P-Value
IRITC ON				
WI	0.397	0.033	12.101	0.000
WD	0.027	0.012	2.212	0.027
COV	-0.036	0.013	-2.857	0.004
HO	-0.005	0.009	-0.553	0.580
SE	-0.193	0.053	-3.670	0.000
RC	-0.078	0.047	-1.663	0.096
SS	-0.203	0.039	-5.227	0.000
JCR	0.088	0.036	2.458	0.014
JCD	-0.082	0.046	-1.795	0.073
WIXJCD	-0.063	0.035	-1.825	0.068

IRITA ON

WI	0.297	0.038	7.866	0.000
WD	0.005	0.013	0.374	0.709
COV	-0.016	0.013	-1.190	0.234
HO	-0.002	0.010	-0.201	0.841
SE	-0.219	0.060	-3.674	0.000
RC	-0.126	0.053	-2.378	0.017
SS	-0.176	0.047	-3.755	0.000
JCR	-0.028	0.040	-0.694	0.488
JCD	0.104	0.049	2.123	0.034
WIXJCD	-0.036	0.045	-0.790	0.430

IRITA WITH

IRITC	0.228	0.032	7.067	0.000

Intercepts

IRITC	2.777	0.033	83.382	0.000
IRITA	2.436	0.036	68.337	0.000

Residual Variances

IRITC	0.754	0.038	19.731	0.000
IRITA	0.866	0.041	21.074	0.000

STANDARDIZED MODEL RESULTS

STDYX Standardization

				Two-Tailed
	Estimate	S.E.	Est./S.E.	P-Value

IRITC ON

WI	0.416	0.033	12.655	0.000
WD	0.082	0.037	2.212	0.027
COV	-0.105	0.037	-2.849	0.004
HO	-0.019	0.035	-0.554	0.580
SE	-0.133	0.036	-3.655	0.000
RC	-0.062	0.037	-1.662	0.096
SS	-0.170	0.033	-5.186	0.000
JCR	0.086	0.035	2.452	0.014
JCD	-0.062	0.034	-1.792	0.073
WIXJCD	-0.055	0.030	-1.822	0.068

IRITA ON

WI	0.311	0.039	8.027	0.000
WD	0.015	0.039	0.374	0.708
COV	-0.046	0.039	-1.189	0.234
HO	-0.008	0.038	-0.200	0.841
SE	-0.151	0.041	-3.666	0.000
RC	-0.100	0.042	-2.375	0.018
SS	-0.146	0.039	-3.759	0.000
JCR	-0.027	0.039	-0.693	0.488
JCD	0.078	0.037	2.126	0.034
WIXJCD	-0.031	0.039	-0.790	0.430

IRITA WITH

IRITC	0.282	0.037	7.672	0.000

Intercepts

IRITC	2.678	0.064	41.989	0.000
IRITA	2.346	0.050	46.623	0.000

Residual Variances

IRITC	0.701	0.029	23.893	0.000
IRITA	0.803	0.028	28.474	0.000

R-SQUARE

Observed Variable	Estimate	S.E.	Two-Tailed Est./S.E.	P-Value
IRITC	0.299	0.029	10.206	0.000
IRITA	0.197	0.028	6.990	0.000

Job crafting - hindering demands x Change in work difficulty

MODEL RESULTS

	Estimate	S.E.	Two-Tailed Est./S.E.	P-Value
IRITC ON				
WI	0.398	0.033	12.123	0.000
WD	0.027	0.012	2.236	0.025
COV	-0.037	0.013	-2.948	0.003
HO	-0.006	0.009	-0.646	0.518
SE	-0.192	0.052	-3.671	0.000
RC	-0.082	0.047	-1.741	0.082
SS	-0.202	0.039	-5.169	0.000
JCR	0.085	0.036	2.352	0.019
JCD	-0.085	0.046	-1.839	0.066
WDXJCD	-0.012	0.014	-0.857	0.391
IRITA ON				
WI	0.297	0.038	7.847	0.000
WD	0.004	0.013	0.322	0.748

COV	-0.016	0.013	-1.254	0.210
HO	-0.003	0.010	-0.281	0.779
SE	-0.217	0.059	-3.654	0.000
RC	-0.126	0.053	-2.383	0.017
SS	-0.172	0.047	-3.661	0.000
JCR	-0.028	0.040	-0.691	0.490
JCD	0.101	0.049	2.049	0.040
WDXJCD	-0.022	0.014	-1.545	0.122

IRITA WITH

IRITC	0.228	0.032	7.112	0.000

Intercepts

IRITC	2.778	0.033	83.409	0.000
IRITA	2.434	0.036	68.475	0.000

Residual Variances

IRITC	0.756	0.039	19.614	0.000
IRITA	0.864	0.041	21.152	0.000

STANDARDIZED MODEL RESULTS

STDYX Standardization

	Estimate	S.E.	Est./S.E.	Two-Tailed P-Value
IRITC ON				
WI	0.417	0.033	12.680	0.000
WD	0.083	0.037	2.237	0.025

COV	-0.109	0.037	-2.940	0.003
HO	-0.023	0.035	-0.646	0.518
SE	-0.132	0.036	-3.656	0.000
RC	-0.065	0.037	-1.740	0.082
SS	-0.169	0.033	-5.131	0.000
JCR	0.082	0.035	2.347	0.019
JCD	-0.064	0.035	-1.836	0.066
WDXJCD	-0.029	0.034	-0.858	0.391

IRITA ON

WI	0.310	0.039	8.010	0.000
WD	0.012	0.038	0.322	0.747
COV	-0.048	0.038	-1.254	0.210
HO	-0.011	0.037	-0.281	0.779
SE	-0.149	0.041	-3.647	0.000
RC	-0.100	0.042	-2.380	0.017
SS	-0.144	0.039	-3.665	0.000
JCR	-0.027	0.039	-0.691	0.490
JCD	0.075	0.037	2.052	0.040
WDXJCD	-0.054	0.035	-1.544	0.123

IRITA WITH

IRITC	0.282	0.037	7.722	0.000

Intercepts

IRITC	2.679	0.063	42.224	0.000
IRITA	2.344	0.051	46.358	0.000

Residual Variances

IRITC	0.703	0.030	23.809	0.000
IRITA	0.801	0.028	28.600	0.000

R-SQUARE

Observed Variable	Estimate	S.E.	Est./S.E.	Two-Tailed P-Value
IRITC	0.297	0.030	10.065	0.000
IRITA	0.199	0.028	7.106	0.000

Job crafting - hindering demands x Risk of being infected by COVID-19

MODEL RESULTS

	Estimate	S.E.	Est./S.E.	Two-Tailed P-Value
IRITC ON				
WI	0.398	0.033	12.093	0.000
WD	0.028	0.012	2.311	0.021
COV	-0.037	0.012	-3.014	0.003
HO	-0.006	0.009	-0.632	0.527
SE	-0.195	0.052	-3.728	0.000
RC	-0.083	0.047	-1.761	0.078
SS	-0.205	0.039	-5.207	0.000
JCR	0.082	0.036	2.265	0.024
JCD	-0.084	0.046	-1.838	0.066
COVXJCD	0.007	0.016	0.432	0.666
IRITA ON				
WI	0.298	0.038	7.852	0.000
WD	0.005	0.013	0.421	0.674
COV	-0.017	0.013	-1.246	0.213
HO	-0.002	0.010	-0.239	0.811
SE	-0.220	0.060	-3.698	0.000

RC	-0.129	0.053	-2.430	0.015
SS	-0.176	0.047	-3.762	0.000
JCR	-0.031	0.040	-0.782	0.434
JCD	0.103	0.049	2.096	0.036
COVXJCD	0.003	0.015	0.214	0.831

IRITA WITH

IRITC	0.230	0.032	7.073	0.000

Intercepts

IRITC	2.779	0.033	83.328	0.000
IRITA	2.438	0.036	68.146	0.000

Residual Variances

IRITC	0.756	0.039	19.618	0.000
IRITA	0.867	0.041	21.152	0.000

STANDARDIZED MODEL RESULTS

STDYX Standardization

				Two-Tailed
	Estimate	S.E.	Est./S.E.	P-Value

IRITC ON

WI	0.417	0.033	12.667	0.000
WD	0.086	0.037	2.312	0.021
COV	-0.110	0.036	-3.005	0.003
HO	-0.022	0.035	-0.633	0.527
SE	-0.134	0.036	-3.710	0.000
RC	-0.066	0.038	-1.760	0.078

SS	-0.171	0.033	-5.169	0.000
JCR	0.079	0.035	2.261	0.024
JCD	-0.063	0.034	-1.834	0.067
COVXJCD	0.017	0.039	0.431	0.666

IRITA ON

WI	0.312	0.039	8.018	0.000
WD	0.016	0.039	0.421	0.674
COV	-0.048	0.039	-1.246	0.213
HO	-0.009	0.038	-0.239	0.811
SE	-0.151	0.041	-3.690	0.000
RC	-0.102	0.042	-2.427	0.015
SS	-0.147	0.039	-3.766	0.000
JCR	-0.030	0.039	-0.781	0.435
JCD	0.077	0.037	2.098	0.036
COVXJCD	0.008	0.036	0.214	0.831

IRITA WITH

IRITC	0.284	0.037	7.697	0.000

Intercepts

IRITC	2.680	0.063	42.416	0.000
IRITA	2.347	0.050	46.624	0.000

Residual Variances

IRITC	0.703	0.029	23.973	0.000
IRITA	0.804	0.028	28.820	0.000

R-SQUARE

Observed			Two-Tailed	
Variable	Estimate	S.E.	Est./S.E.	P-Value

IRITC	0.297	0.029	10.108	0.000
IRITA	0.196	0.028	7.035	0.000

Job crafting - hindering demands x Change in home office

MODEL RESULTS

			Two-Tailed	
	Estimate	S.E.	Est./S.E.	P-Value
IRITC ON				
WI	0.398	0.033	12.106	0.000
WD	0.028	0.012	2.297	0.022
COV	-0.037	0.013	-2.944	0.003
HO	-0.006	0.009	-0.644	0.520
SE	-0.193	0.052	-3.708	0.000
RC	-0.084	0.047	-1.767	0.077
SS	-0.204	0.039	-5.186	0.000
JCR	0.083	0.036	2.317	0.020
JCD	-0.084	0.046	-1.818	0.069
HOXJCD	0.003	0.012	0.219	0.826
IRITA ON				
WI	0.298	0.038	7.887	0.000
WD	0.005	0.013	0.393	0.694
COV	-0.016	0.013	-1.217	0.224
HO	-0.002	0.010	-0.181	0.856
SE	-0.221	0.060	-3.700	0.000
RC	-0.129	0.053	-2.426	0.015
SS	-0.177	0.047	-3.782	0.000
JCR	-0.030	0.040	-0.761	0.446
JCD	0.103	0.049	2.089	0.037

HOXJCD	-0.006	0.012	-0.517	0.605

IRITA WITH

IRITC	0.230	0.032	7.092	0.000

Intercepts

IRITC	2.781	0.034	82.902	0.000
IRITA	2.436	0.036	67.323	0.000

Residual Variances

IRITC	0.757	0.039	19.598	0.000
IRITA	0.867	0.041	21.178	0.000

STANDARDIZED MODEL RESULTS

STDYX Standardization

	Estimate	S.E.	Est./S.E.	Two-Tailed P-Value
IRITC ON				
WI	0.418	0.033	12.676	0.000
WD	0.085	0.037	2.299	0.022
COV	-0.109	0.037	-2.937	0.003
HO	-0.022	0.035	-0.644	0.519
SE	-0.133	0.036	-3.692	0.000
RC	-0.066	0.038	-1.766	0.077
SS	-0.171	0.033	-5.148	0.000
JCR	0.081	0.035	2.312	0.021
JCD	-0.063	0.035	-1.816	0.069

HOXJCD	0.008	0.037	0.219	0.826

IRITA ON

WI	0.312	0.039	8.057	0.000
WD	0.015	0.039	0.393	0.694
COV	-0.047	0.038	-1.216	0.224
HO	-0.007	0.038	-0.181	0.856
SE	-0.152	0.041	-3.694	0.000
RC	-0.102	0.042	-2.422	0.015
SS	-0.148	0.039	-3.787	0.000
JCR	-0.029	0.039	-0.761	0.447
JCD	0.077	0.037	2.091	0.036
HOXJCD	-0.019	0.036	-0.517	0.605

IRITA WITH

IRITC	0.284	0.037	7.716	0.000

Intercepts

IRITC	2.682	0.063	42.299	0.000
IRITA	2.345	0.051	46.142	0.000

Residual Variances

IRITC	0.704	0.030	23.806	0.000
IRITA	0.804	0.028	28.804	0.000

R-SQUARE

Observed Variable	Estimate	S.E.	Est./S.E.	Two-Tailed P-Value
IRITC	0.296	0.030	10.027	0.000
IRITA	0.196	0.028	7.044	0.000

Multi-group analysis

MODEL RESULTS

	Estimate	S.E.	Est./S.E.	Two-Tailed P-Value

Group GERMANY

IRITC ON				
WI	0.483	0.063	7.666	0.000
WD	0.042	0.026	1.656	0.098
COV	-0.049	0.022	-2.272	0.023
HO	0.015	0.019	0.779	0.436
SE	-0.250	0.097	-2.577	0.010
RC	-0.004	0.092	-0.039	0.969
SS	-0.299	0.093	-3.219	0.001
JCR	0.129	0.069	1.889	0.059
JCD	0.022	0.097	0.226	0.821

IRITA ON				
WI	0.433	0.074	5.828	0.000
WD	-0.025	0.025	-1.020	0.307
COV	-0.036	0.025	-1.409	0.159
HO	-0.025	0.021	-1.210	0.226
SE	-0.238	0.116	-2.050	0.040
RC	-0.191	0.102	-1.874	0.061
SS	-0.249	0.110	-2.262	0.024
JCR	0.017	0.079	0.213	0.831
JCD	0.151	0.096	1.567	0.117

IRITA WITH

IRITC	0.196	0.065	3.017	0.003

Intercepts

IRITC	2.781	0.075	37.222	0.000
IRITA	2.503	0.082	30.663	0.000

Residual Variances

IRITC	0.620	0.069	8.950	0.000
IRITA	0.773	0.095	8.158	0.000

Group CZECHIA

IRITC ON

WI	0.354	0.058	6.081	0.000
WD	0.038	0.022	1.709	0.087
COV	-0.039	0.024	-1.630	0.103
HO	0.021	0.015	1.394	0.163
SE	-0.123	0.083	-1.484	0.138
RC	-0.234	0.073	-3.194	0.001
SS	-0.186	0.064	-2.917	0.004
JCR	0.137	0.058	2.383	0.017
JCD	-0.309	0.075	-4.115	0.000

IRITA ON

WI	0.261	0.063	4.160	0.000
WD	0.014	0.022	0.620	0.536
COV	0.000	0.025	-0.008	0.993
HO	0.020	0.016	1.243	0.214
SE	-0.158	0.093	-1.708	0.088
RC	-0.206	0.077	-2.686	0.007
SS	-0.189	0.085	-2.233	0.026
JCR	0.007	0.061	0.119	0.905
JCD	0.082	0.082	0.997	0.319

IRITA WITH

IRITC	0.208	0.049	4.243	0.000

Intercepts

IRITC	2.841	0.058	48.624	0.000
IRITA	2.497	0.063	39.485	0.000

Residual Variances

IRITC	0.712	0.069	10.253	0.000
IRITA	0.775	0.059	13.214	0.000

Group SLOVAKIA

IRITC ON

WI	0.329	0.064	5.135	0.000
WD	0.007	0.027	0.245	0.807
COV	-0.025	0.030	-0.822	0.411
HO	-0.012	0.022	-0.539	0.590
SE	-0.212	0.108	-1.969	0.049
RC	0.009	0.109	0.080	0.936
SS	-0.128	0.084	-1.534	0.125
JCR	0.029	0.089	0.325	0.746
JCD	0.025	0.102	0.245	0.806

IRITA ON

WI	0.247	0.074	3.332	0.001
WD	0.031	0.028	1.112	0.266
COV	-0.027	0.028	-0.976	0.329
HO	-0.009	0.022	-0.417	0.677
SE	-0.287	0.124	-2.316	0.021
RC	0.070	0.123	0.567	0.571

SS	-0.169	0.096	-1.762	0.078
JCR	-0.070	0.097	-0.724	0.469
JCD	0.051	0.112	0.455	0.649

IRITA WITH

IRITC	0.293	0.071	4.133	0.000

Intercepts

IRITC	2.781	0.080	34.840	0.000
IRITA	2.519	0.085	29.602	0.000

Residual Variances

IRITC	0.862	0.082	10.471	0.000
IRITA	0.919	0.092	10.011	0.000

Group ITALY

IRITC ON

WI	0.469	0.062	7.549	0.000
WD	0.021	0.022	0.961	0.336
COV	-0.029	0.019	-1.553	0.120
HO	-0.052	0.018	-2.820	0.005
SE	-0.355	0.122	-2.899	0.004
RC	-0.037	0.089	-0.411	0.681
SS	-0.146	0.064	-2.262	0.024
JCR	-0.041	0.083	-0.497	0.619
JCD	-0.013	0.095	-0.134	0.893

IRITA ON

WI	0.275	0.077	3.564	0.000
WD	0.038	0.027	1.392	0.164
COV	0.008	0.026	0.310	0.757
HO	0.011	0.025	0.453	0.651

SE	-0.283	0.147	-1.927	0.054
RC	-0.175	0.119	-1.462	0.144
SS	-0.148	0.092	-1.616	0.106
JCR	-0.014	0.097	-0.140	0.889
JCD	0.041	0.117	0.348	0.728

IRITA WITH

IRITC	0.168	0.064	2.634	0.008

Intercepts

IRITC	2.824	0.078	36.061	0.000
IRITA	2.160	0.100	21.645	0.000

Residual Variances

IRITC	0.615	0.065	9.472	0.000
IRITA	0.896	0.085	10.532	0.000

STANDARDIZED MODEL RESULTS

STDYX Standardization

	Estimate	S.E.	Est./S.E.	Two-Tailed P-Value

Group GERMANY

IRITC ON

WI	0.479	0.062	7.704	0.000
WD	0.124	0.073	1.682	0.092
COV	-0.138	0.062	-2.219	0.026

HO	0.051	0.066	0.777	0.437
SE	-0.172	0.065	-2.626	0.009
RC	-0.003	0.065	-0.039	0.969
SS	-0.239	0.078	-3.067	0.002
JCR	0.124	0.066	1.891	0.059
JCD	0.014	0.064	0.226	0.821

IRITA ON

WI	0.431	0.074	5.825	0.000
WD	-0.075	0.074	-1.014	0.310
COV	-0.100	0.071	-1.413	0.158
HO	-0.087	0.072	-1.209	0.226
SE	-0.165	0.080	-2.074	0.038
RC	-0.135	0.073	-1.846	0.065
SS	-0.200	0.090	-2.235	0.025
JCR	0.016	0.076	0.213	0.831
JCD	0.099	0.063	1.567	0.117

IRITA WITH

IRITC	0.283	0.086	3.277	0.001

Intercepts

IRITC	2.562	0.129	19.913	0.000
IRITA	2.319	0.113	20.455	0.000

Residual Variances

IRITC	0.527	0.058	9.127	0.000
IRITA	0.664	0.068	9.740	0.000

Group CZECHIA

IRITC ON

WI	0.364	0.059	6.144	0.000

WD	0.110	0.064	1.721	0.085
COV	-0.105	0.065	-1.621	0.105
HO	0.078	0.056	1.400	0.161
SE	-0.086	0.058	-1.477	0.140
RC	-0.195	0.062	-3.159	0.002
SS	-0.141	0.049	-2.886	0.004
JCR	0.139	0.059	2.360	0.018
JCD	-0.232	0.057	-4.058	0.000

IRITA ON

WI	0.279	0.066	4.253	0.000
WD	0.042	0.067	0.620	0.535
COV	-0.001	0.069	-0.008	0.993
HO	0.081	0.065	1.248	0.212
SE	-0.115	0.067	-1.709	0.087
RC	-0.179	0.066	-2.706	0.007
SS	-0.149	0.068	-2.211	0.027
JCR	0.008	0.064	0.119	0.905
JCD	0.064	0.064	0.996	0.319

IRITA WITH

IRITC	0.280	0.060	4.662	0.000

Intercepts

IRITC	2.789	0.102	27.322	0.000
IRITA	2.549	0.091	28.004	0.000

Residual Variances

IRITC	0.686	0.054	12.643	0.000
IRITA	0.808	0.046	17.701	0.000

Group SLOVAKIA

IRITC ON

WI	0.384	0.072	5.351	0.000
WD	0.019	0.077	0.245	0.807
COV	-0.074	0.090	-0.822	0.411
HO	-0.044	0.081	-0.540	0.589
SE	-0.164	0.085	-1.934	0.053
RC	0.007	0.088	0.080	0.936
SS	-0.110	0.071	-1.542	0.123
JCR	0.027	0.084	0.325	0.745
JCD	0.019	0.076	0.245	0.806

IRITA ON

WI	0.284	0.085	3.340	0.001
WD	0.088	0.079	1.113	0.266
COV	-0.079	0.081	-0.974	0.330
HO	-0.034	0.081	-0.417	0.677
SE	-0.218	0.094	-2.309	0.021
RC	0.056	0.098	0.568	0.570
SS	-0.143	0.081	-1.766	0.077
JCR	-0.065	0.089	-0.724	0.469
JCD	0.037	0.081	0.455	0.649

IRITA WITH

IRITC	0.329	0.069	4.797	0.000

Intercepts

IRITC	2.673	0.135	19.854	0.000
IRITA	2.386	0.110	21.641	0.000

Residual Variances

IRITC	0.797	0.055	14.607	0.000
IRITA	0.825	0.059	14.020	0.000

Group ITALY

IRITC ON				
WI	0.461	0.055	8.330	0.000
WD	0.062	0.065	0.952	0.341
COV	-0.096	0.062	-1.540	0.124
HO	-0.196	0.068	-2.871	0.004
SE	-0.200	0.068	-2.926	0.003
RC	-0.029	0.071	-0.409	0.683
SS	-0.137	0.061	-2.257	0.024
JCR	-0.038	0.076	-0.498	0.618
JCD	-0.009	0.070	-0.134	0.893
IRITA ON				
WI	0.258	0.070	3.696	0.000
WD	0.105	0.075	1.398	0.162
COV	0.026	0.083	0.310	0.757
HO	0.042	0.092	0.452	0.651
SE	-0.152	0.081	-1.885	0.059
RC	-0.132	0.090	-1.473	0.141
SS	-0.133	0.081	-1.640	0.101
JCR	-0.012	0.085	-0.140	0.889
JCD	0.028	0.082	0.347	0.728
IRITA WITH				
IRITC	0.226	0.086	2.613	0.009
Intercepts				
IRITC	2.806	0.163	17.243	0.000
IRITA	2.052	0.124	16.519	0.000

Residual Variances

IRITC	0.608	0.058	10.516	0.000
IRITA	0.808	0.054	14.950	0.000

R-SQUARE

Group GERMANY

Observed Variable	Estimate	S.E.	Two-Tailed Est./S.E.	P-Value
IRITC	0.473	0.058	8.206	0.000
IRITA	0.336	0.068	4.935	0.000

Group CZECHIA

Observed Variable	Estimate	S.E.	Two-Tailed Est./S.E.	P-Value
IRITC	0.314	0.054	5.793	0.000
IRITA	0.192	0.046	4.208	0.000

Group SLOVAKIA

Observed Variable	Estimate	S.E.	Two-Tailed Est./S.E.	P-Value
IRITC	0.203	0.055	3.725	0.000
IRITA	0.175	0.059	2.981	0.003

Group ITALY

Observed Variable	Estimate	S.E.	Est./S.E.	Two-Tailed P-Value
IRITC	0.392	0.058	6.786	0.000
IRITA	0.192	0.054	3.554	0.000

. The figure legends are required to have the same font as the main text, 12 point normal Times New Roman, single spaced. Please use a single paragraph for each legend and prepare the figures keeping in mind the PDF layout.
